# Microtubule regulation in cancer cells

**DOI:** 10.3389/fcell.2025.1677302

**Published:** 2026-08-21

**Authors:** Alexandre Matov

**Affiliations:** DataSet Analysis LLC, San Francisco, CA, United States

**Keywords:** spindle rotation, breast cancer, prostate cancer, microtubule dynamics, chromosome segregation, tubulin acetylation, circulating tumor cells, patient-derived organoids

## Abstract

**Introduction:**

The notion of microtubule (MT) dynamics relates to the changes in the length of MT polymers in living cells. They are governed by a stochastic process related to the rates and chances of adding or removing tubulin dimers at the ends of MT polymers, termed dynamic instability. The ability of each MT to swiftly switch between stages of adding or removing dimers at the tip of its lattice is critical for the overall success of the mitotic spindle, a molecular machinery built dynamically by MTs and associated molecular motor proteins, in properly segregating the duplicated DNA into the two daughter cells. When changes in the genetic and epigenetic regulation of the cell affect this ability, for example, by increasing the rates of hydrolysis of bound to tubulin dimers incorporated in the MT lattice, that results in segregation errors and is a hallmark of disease.

**Methods:**

In cancer, the dysregulation of MT dynamics contributes to drug resistance. Measuring and modeling MT dynamics provides an insight into the regulation of the cell and its susceptibility to drug action. It can also characterize the function of patient immune cells and contribute to improving the success rate of cell therapy.

**Results:**

Our investigation indicates that drug-resistant tumors exhibit particular changes in their regulation at the cellular level that expose cell state vulnerabilities, which can be targeted during therapy. We elucidate mechanisms of oncogenic activity in dividing cells and propose an approach for overcoming drug resistance.

**Conclusion:**

Changes in MT regulation before and after drug treatment of patient cells are indicative of the susceptibility of tumors to particular drug regimens.

## Introduction

The MT is an elongated (Inoué, 1959), hollow polymer (Porter, 1963) consisting of 13 protofilaments wrapped into a cylinder (Tilney et al., 1973). The dynamic addition or removal of dimers at the ends of MT polymers is termed dynamic instability (Mitchison and Kirschner, 1984), a process that is critical for the success of the mitotic spindle in segregating the duplicated DNA into the two daughter cells without errors (Nasmyth, 2002). Tubulin inhibitors that stabilize MTs, like the taxanes (Wani et al., 1971), crawl inside the hollow MT and bind to the luminal side of its lattice (Nogales et al., 1998). This way, the ability of the MT to depolymerize is inhibited (Derry et al., 1995). The MT continues polymerizing, albeit with reduced rates (Derry et al., 1995), but can no longer switch to a state of depolymerization because of the drug molecules bound inside of it. Eventually, when the drug reaches a high concentration, the cell forms unusually long MTs that cluster next to each other, forming thick MT bundles (Kowalski et al., 1997; Manfredi et al., 1982; Matov, 2024d). If this happens in patient tumor cells, it would be associated with a significant toxicity and considerable side effects (Cella et al., 2003).

For instance, median docetaxel plasma levels of 3 nM is reached 72 h after a dose of 20 mg/m^2^ (Brunsvig et al., 2007). This dose is 5-fold lower than the 3-weekly treatment with 100 mg/m^2^ docetaxel in breast cancer (BC) (Jones et al., 2005) and such high levels of the drug kill many plasma neutrophils and lymphocytes, where the drug accumulates. High doses of traditional tubulin inhibitors result in, besides the side effects, the development of other neoplasms, such as myeloid neoplasms (Bhatnagar et al., 2016; Gupta et al., 2014), or/and neurodegenerative diseases like dementia (Ren et al., 2017). Low doses of the drug could efficiently kill tumor cells that are susceptible to its action either because of a particular tumor vulnerability (Linn et al., 1994; Vecchione et al., 2016) or as a result of a prior sensitizing treatment with another drug (Mertens et al., 2023). Alternatively, a low dose treatment with a tubulin inhibitor could sensitize the tumors to the cellular effects of small molecules that exploit cancer cell vulnerabilities (Hangauer et al., 2017). We explore several cellular features of oncogenic activity leading to resistant phenotypes that cannot be addressed by increasing the drug dose, but can likely be overcome via a drug combination selected based on the analysis of cytoskeletal modulation. There exist common outliers in the regulation of subsets of dividing cells associated with oncogenic activity across epithelial cells of different origin. The regulation of MTs at their minus ends is particularly affected in tumor cells (Malumbres and Barbacid, 2007) and cells infected by a virus (Ploubidou et al., 2000), resulting in a pendulum-like rotation of the mitotic spindle (Matov, 2024f). Posttranslational modifications (Cleveland and Sullivan, 1985) might be contributing factors in such cells.

The modulation of MT dynamics could be utilized as a quantitative readout of the effects induced by regimens targeting RAS. *Ras* genes (the *HRas*, *KRas*, and *NRas* genes) are the most frequently mutated oncogenes in human cancer (Bos, 1989; Zehir et al., 2017). RAS proteins belong to the RAS superfamily of at least five families. They regulate a wide variety of cellular functions as biological timers that initiate and terminate specific functions and determine the periods of time for the continuation of the specific cellular functions. They furthermore play key roles in not only temporal but also spatial regulation of specific functions. The RAS family regulates gene expression, the Rho family regulates cytoskeletal organization and gene expression, the Rab and ARF families regulate vesicle trafficking, and the Ran family regulates nucleocytoplasmic transport and MT organization (Bourne et al., 1990; Takai et al., 2001). RAS small GTPases act as molecular switches, cycling between an inactive GDP-bound (OFF) state and an active GTP-bound (ON) state. The exchange of GDP for GTP induces a conformational change in the proteins that allows them to interact with their downstream effectors and carry out their multiple functions (Rodriguez-Viciana et al., 2004). One of the best characterized RAS effectors are the RAF kinases, through which RAS activates the MAPK pathway (Ehrhardt et al., 2002). MAPKs play crucial roles in cell migration by distinct mechanisms, for instance by phosphorylating focal adhesion (FA) proteins and MT-associated proteins (MAPs) (Huang et al., 2004).

We have shown that in renal cell carcinoma cells MT polymerization rates exhibit a bimodal distribution (Matov et al., 2006). There are significant differences between fast polymerizing (25.7 μm/min ± 2.9) and slow polymerizing (6.7 μm/min ± 12.2) MTs in *VHL* ^*−/−*^ cells. Upon reconstitution of pVHL, only one population of slow polymerizing (8.2 μm/min ± 5.1) MTs remained (Matov, 2006; Matov et al., 2006). Exponentially-growing renal cell carcinoma cells producing the phosphorylation site mutant pVHL30(S68/72A) that is resistant to phosphorylation by GSK3β and the phosphorylation-mimicking mutant pVHL30(S68/72D) exhibit similar rates of MT polymerization (Matov et al., 2006). The MT polymerization rates are, however, significantly increased in serum-starved cells that expressed pVHL30(S68/72D) (suggesting a loss of pVHL function) but not in cells expressing pVHL30(S68/72A) (Matov, 2024d). Biochemically, these effects on the MT dynamics are mediated by pVHL, which reduces intrinsic GTPase activity and the hydrolysis of GTP-bound tubulin. *In vitro*, in the presence of active pVHL30, the rate of tubulin GTP hydrolysis was lower – similarly, in cells with active pVHL30, we measured higher frequencies of GTP-capped MT plus ends and GTP remnants along the MT lattice (Thoma et al., 2010).

Oncogenic RAS remains in a GTP-bound state and drugs that target RAS have been aimed at restoring the ability of the protein to hydrolyze GTP to GDP (Cuevas-Navarro et al., 2025). To become active then, RAS would need to recruit GTP molecules. RAS activity reduces the rates of MT polymerization and nucleation by activating the MAPK/ERK pathway (Hoshi et al., 1992). In hypoxic cells, MAPK increases the phosphorylation of MAP4 and decreases the phosphorylation of Op18, which leads to a sparser MT network and frequent MT depolymerization (Hu et al., 2010). The effects on MT dynamics of RAS activity seem similar to the loss of pVHL, which we have shown to increase MT depolymerization rates and the MT catastrophe frequencies (that is, the rates with which MTs switch from polymerization to depolymerization) (Thoma et al., 2010). Therefore, quantitative live-cell image analysis of MT dynamics can be utilized to evaluate the activity of RAS-targeting drugs in patient cells *ex vivo*.

RIT1 (Wes et al., 1996), a RAS-related GTPase, can activate RAF (Rodriguez-Viciana et al., 2004) and its targeting results in tumor shrinkage in mouse models of cancer (Mozzarelli et al., 2025). Because RIT1 translocation to the plasma membrane during anaphase is essential for timely progression through mitosis and proper chromosome segregation (Cuevas-Navarro et al., 2021), image analysis of dividing cells can help evaluate RIT1-targeting drugs. RAS tri-complex inhibitors (TCIs) bind to GTP-bound (ON) RAS in complex with cyclophilin A (CYPA) and inhibit its activity (Schulze et al., 2023). CYPA is involved in multiple cellular processes, such as protein folding, intracellular trafficking, signal transduction, and transcriptional regulation (Xue et al., 2018). It is transported by cytoplasmic dynein along MTs toward their minus ends (Galigniana et al., 2004) and image analysis of MTs in living cells can elucidate mechanisms of resistance to TCIs.

## Results

### Simian virus 40 causes aneuploidy in breast epithelial cells

#### Knockdown of MYC does not affect microtubule regulation in dividing MDA-MB-231 cells

Aggressive forms of BC, such as receptor triple-negative cancers (TNBCs), are associated with worse patient outcomes (Maqbool et al., 2022). Although taxanes are initially effective in reducing tumor burden for some patients, resistance to MT-targeting systemic therapy is universal and the cell biological profiling of patient-derived cells *ex vivo* is not utilized as a rationale for identifying drug-sensitive and drug-resistant patient populations prior to initiating therapy. The signaling molecules that interact with MTs, as well as the multiple effects on signaling pathways that destabilize or hyperstabilize MTs, indicate that MTs are likely to be critical to the spatial organization of signal transduction. In receptor TNBC MDA-MB-231 cells, we identified the formation of larger spindles with high MT density (Matov, 2024f) and measured very rapid lateral twisting during metaphase that is distinct from other cell lines. We reported quantitative measurements of these oscillations and the corresponding waves of alterations of end binding protein 1 (EB1) comet speeds (Matov et al., 2015) as a possible mechanism of spindle positioning that is disrupted in breast tumors resistant to treatment with tubulin inhibitors. The *Myc* oncogene is overexpressed in MBA-MD-231 cells (Horiuchi et al., 2012) and MYC has been shown to induce tubulin acetylation and alter cell morphology, because longer MTs form (Conacci-Sorrell et al., 2010). When implanted in immunodeficient mice, MBA-MD-231 cells do not migrate and do not form metastatic tumors if MYC is knocked down (Wolfer et al., 2010), likely due to changes in the regulation of filamentous actin (F-actin) (Matov, 2024e; Matov, 2024i; Matov, 2025b). We imaged dozens of dividing MBA-MD-231 cells after knockdown of MYC (MYC siRNA (Rohrberg et al., 2020)) to investigate whether the loss of MYC would reduce spindle size, the spindle rapid motion, and lateral rotation – all of which remained completely unaffected (Vid. 1 and Vid. 2), even after the addition of a B-RAF or an EGFR inhibitor (Matov, 2024i). Our new results indicate that the knockdown of MYC does not affect MT regulation in dividing MDA-MB-231 cells.

#### Expression of the simian virus 40 large T antigen increases microtubule polymerization in dividing cells

**FIGURE 1:**
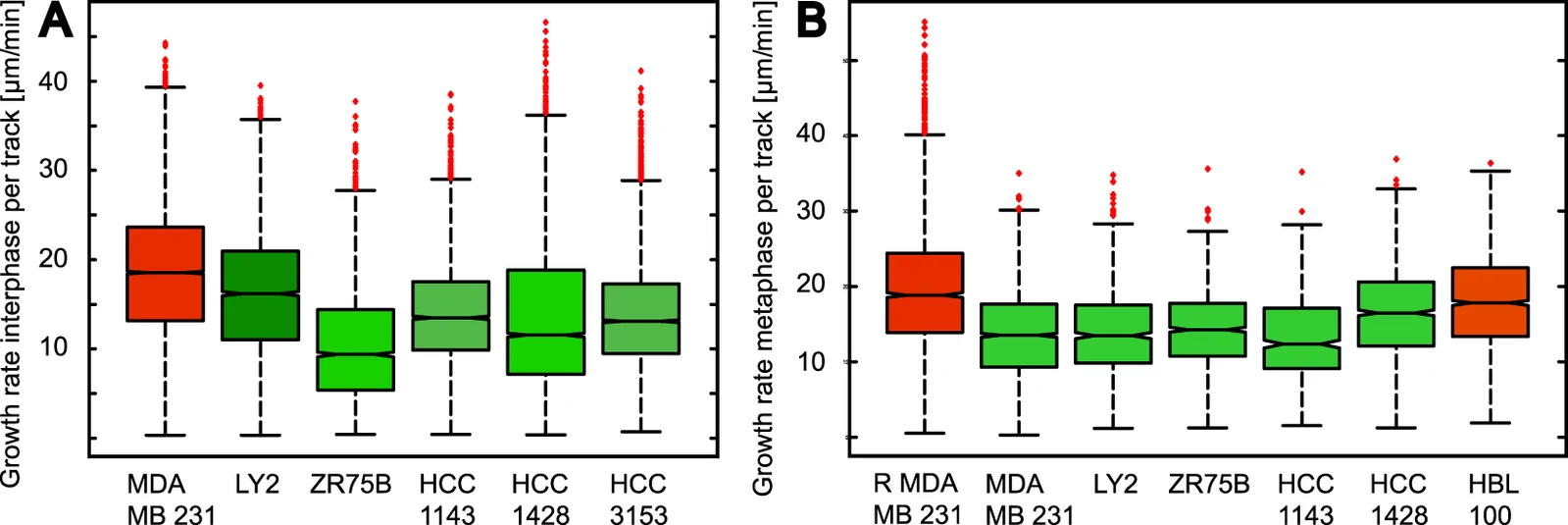
MT plus tip dynamics in BC cell lines. **(A)** MDA-MB-231 TNBC cells have elevated rates of MT polymerization in interphase. ZR-75-B ER-positive cells have the lowest rates of MT polymerization rates in interphase. **(B)** In metaphase, most BC cell lines exhibit similar EB1 speeds. We have uncoupled “rotating” and “not rotating” spindles for the MDA-MB-231 cell line. Surprisingly, HBL-100 cells display the most elevated rates of polymerization (when we exclude the rotating component of the MDA-MB-231 cells). HBL-100 are breast epithelial cells that express SV40 large T antigen. See Supplementary Table S1 for the number of videos, tracks, and further statistics.

In interphase MDA-MB-231 cells, MTs exhibit the fastest polymerization rates, significantly faster than any other BC cell line (Figure 1A). During metaphase, however, after splitting the MDA-MD-231 spindles into “rotating” and “not rotating”, we measured very uniform polymerization rates across cell lines and cells with not rotating spindles – with the notable exception of the HBL-100 spindles (Figure 1B). All EB1 comet speeds were measured in HBL-100 spindles that are not rotating (see also Supplementary Table S1), just slightly rocking in some cases, and therefore the elevated MT tip speeds are indicative of an enhanced MT polymerization activity with a very little spindle rotation component. The HBL-100 cell line is peculiar in the sense that it is established from normal breast epithelium after spontaneous immortalization and exhibits characteristics of neoplastic cells, such as aneuploidy and continuous growth (Gaffney, 1982). The HBL-100 cells have been shown to express the Simian virus 40 (SV40) large T antigen, which significantly affects the biology of the cell (Caron de Fromentel et al., 1985). During infection, SV40 is disassembled in the cytosol and trafficked to the nucleus by cytoplasmic dynein (Ravindran et al., 2018). SV40 large T antigen has been shown to bind TACC2 and induce the formation of disorganized mitotic spindles, slow progression of mitosis, and chromosome segregation errors (Tei et al., 2009). TACC2 is strongly concentrated at centrosomes throughout the cell cycle (Gergely et al., 2000) and its targeting by SV40 during mitosis reduces the ability of the spindle to focus the spindle poles. SV40 large T antigen causes a delay in the progression to anaphase by reducing the tension between kinetochores and leads to the formation of micronuclei (Hu et al., 2013). Our new results indicate that the expression of the SV40 causes aneuploidy in breast epithelium.

#### HBL-100 breast epithelial spindles exhibit hallmarks of cancer

Besides the elevated MT polymerization rates during cell division, we identified three types of abnormal spindle behavior in dozens of HBL-100 cells. Similarly to the rotation of the spindle in the most aggressive MDA-MB-231 TNBC cells, the expression of SV40 large T antigen in HBL-100 cells has induced a rocking spindle motion as well (Vid. 3). Further, SV40 expression induced the formation of misplaced spindles (that is, not being located in the center of the dividing cell (Thoma et al., 2009)), also a hallmark of cancer and common in the TNBC MDA-MB-231 cells (Vid. 4). In some spindles, SV40 expression induced the formation of unfocused spindle poles (Goshima et al., 2005; Zong et al., 2016), a hallmark of cancer (Vid. 5).

Rapid motions of the spindle poles correlated with MT-cortex interactions and bending of MTs suggested pushing forces underlying these spindle rotations. Integrin signaling regulates spindle orientation in a ligand independent manner (Petridou and Skourides, 2016) and E-cadherin re-expression suppresses the development of osteolytic bone metastases in mice bearing the mesenchymal MDA-MB-231 cells (Mbalaviele et al., 1996). We showed that in lung cancer patient urine samples there are low abundance levels of microRNA-532–3p (Matov, 2024g; Matov, 2024k), which regulates the kinesin KIFC1/HSET – known to play a critical role in centrosome clustering – a protein that is overexpressed in TNBC and promotes tumor progression via different mechanisms (Li Y. et al., 2015; Pannu et al., 2015).

Importantly, the astral MTs in rotating spindles are unusually long and seem to have lost their ability to depolymerize, and thus shorten, in order to position (Hertwig, 1884) the spindle – a process driven by adhesion to actin binding proteins (Kavallaris, 2010; Matov, 2024e; Théry et al., 2005), such as dynactin, in proximity to the poles (Bergstralh et al., 2017). The longer this rocking motion continues, the less likely it becomes for the astral MTs to undergo catastrophe because of posttranslational modifications (Cleveland and Sullivan, 1985), such as tubulin acetylation (Matov et al., 2010). Taken together, our new results indicate that the expression of the SV40 induces multiple hallmarks of cancer in breast epithelial cells.

#### Overexpression of MYC reduces spindle microtubule numbers in RPE cells

Overexpression of the *Myc* oncogene has been linked to the genesis of a plethora of human tumors (Felsher and Bishop, 1999). Paradoxically, *Myc* can also act as a suppressor of cell motility, invasiveness, and metastasis by repressing the transcription of integrins (Liu et al., 2012), which implies a reduced formation of FAs. Rap1 regulates cell adhesion (Bos et al., 2001) and cell motility through the regulation of myosin II, and activated Rap1 is preferentially found at the leading edge of chemotaxing cells (Jeon et al., 2007). Rap1 and RhoA GTPase signaling reduces the size and inhibits the sliding of FAs in prostate cells (Spanjaard et al., 2015), which mimic the effects of inhibition of RhoA-mediated cytoskeletal contractility and promotes metastasis (Bailey et al., 2009). Cancer cells can switch between Rho/ROCK-mediated (Matov, 2024i) contractility-based motility and Rac1-mediated (Matov, 2025a) mesenchymal migration. MTs locally modulate the activity of Rho GTPases, which initiate the polarization of the MT cytoskeleton in migrating cells (Wittmann and Waterman-Storer, 2001), whereas Rac1 has been shown to decrease MT catastrophe frequency and regulate MT dynamics at the leading edge (Wittmann et al., 2003).

CLASPs preferentially bind the MT plus ends in the cell body, but bind along the entire MT lattice – a process regulated by Rac1 – in the lamella and lamellipodium (Wittmann and Waterman-Storer, 2005), which inhibits MT catastrophe at the leading edge. Our analysis has demonstrated that the density of polymerizing MTs is highest in the adhesion zone and reaches its peak at 3.5 µm from the cell edge. At distances of less than 3.5 µm to the cell edge, MT density is reduced (Matov et al., 2005) and their rates of polymerization are attenuated with 3.2 μm/min (Matov et al., 2006). Loss of Rap1GAP suppresses ROCK-mediated contractility and, instead, Rac1-mediated contractility enhances cell migration velocity and directionality (Tsygankova et al., 2013). FAs (Couchman and Rees, 1979) are tension sensitive structures that orchestrate cell migration (Winograd-Katz et al., 2014). Their size and maturation are controlled by vinculin (Adams et al., 2004) and the contractility of the connected acto-myosin cytoskeleton. When contractility is reduced by partial inhibition of myosin II activity, the FAs shrink and eventually – once contractility is inhibited – disappear (Pasapera et al., 2010).

**FIGURE 2:**
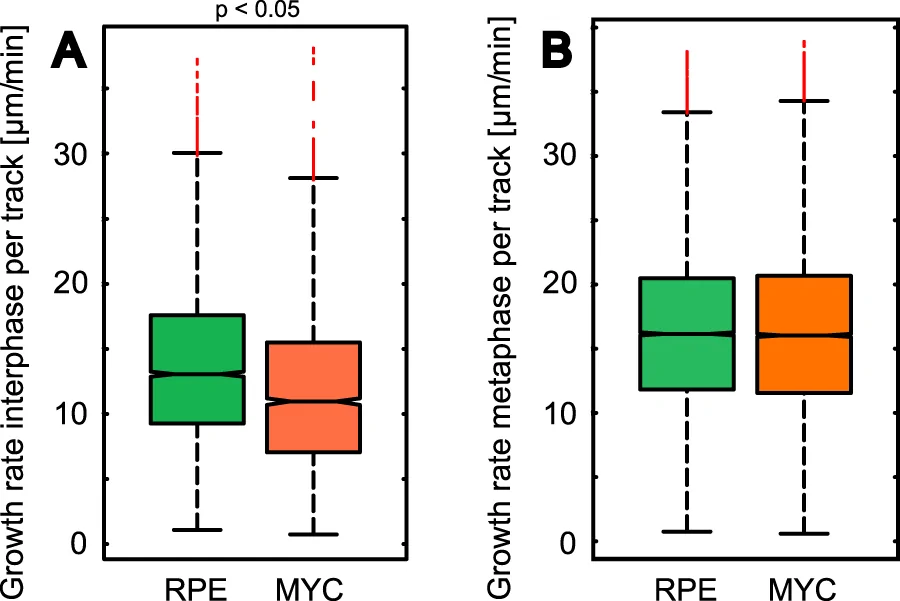
Effects of overexpression of MYC per MT. **(A)** MT growth rates are attenuated with 16% during interphase in RPE cells when MYC is overexpressed. See also Supplementary Table S1. **(B)** The speeds of individual EB1 comets remain unattenuated during metaphase in RPE cells when MYC is overexpressed. During mitosis, MYC leads to the formation of 30% less EB1 comets, but they move with the same speeds. See Supplementary Table S2 for the number of videos, tracks, and further statistics.

**FIGURE 3:**
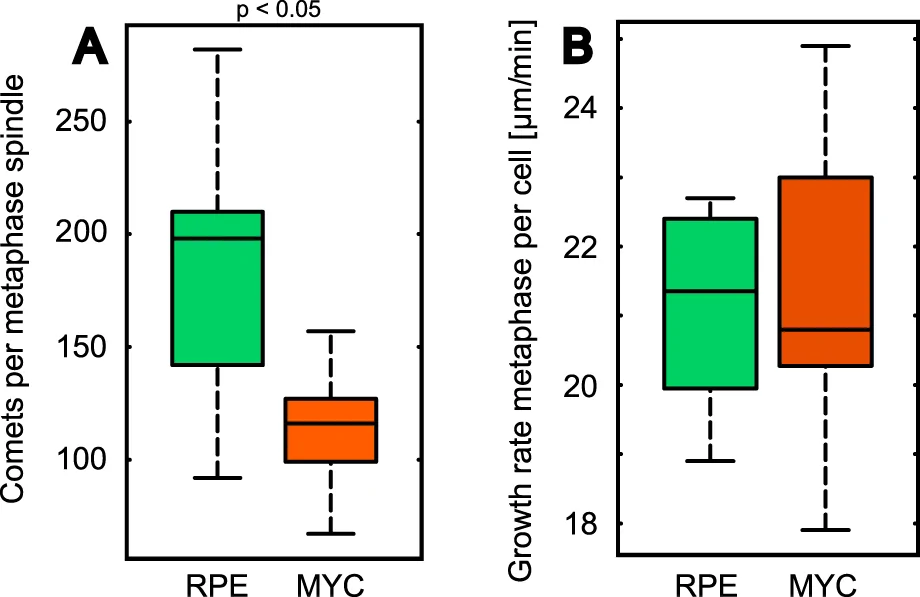
Effects of overexpression of MYC per cell. **(A)** The number of polymerizing MTs is reduced with 38% during metaphase in RPE cells when MYC is overexpressed. **(B)** The average MT growth rates remain unattenuated during metaphase in RPE cells when MYC is overexpressed. Overexpression of MYC leads to less than 2% median decrease of MT polymerization rates per cell, not statistically significant. See Supplementary Table S2 for the number of videos, tracks, and further statistics.

To study the changes in MT dynamics (Salmon et al., 1984; Saxton et al., 1984) *Myc* overexpression causes, we measured EB1 speeds in retinal pigment epithelial cells, which do not express MYC, and compared them to cells that overexpress MYC (RPE-MYC (Goga et al., 2007)). MYC overexpression did not affect MT polymerization rates in metaphase cells and mildly attenuated them in interphase cells (Figure 2; Supplementary Table S1). Interestingly, MYC overexpression reduced the number of polymerizing MTs by 30% during mitosis, which was the result of both the formation of sparser MT bundles and smaller spindles (Figure 2, Figure 3; Supplementary Table S2, and Vid. 6). The interphase cells with overexpressed MYC were also distinctly smaller (Vid. 7), which is a hallmark of drug resistance (Matov, 2024d). Branching MTs nucleation, which is frequent in the spindle, is mediated by augmin and TPX2 (Wittmann et al., 1998; Wittmann et al., 2000), and the upregulation of TPX2 leads to the formation of very dense MT patterns (Petry et al., 2013). It is conceivable that overexpression of MYC downregulates TPX2, which would also affect the levels of Aurora A (Eyers et al., 2003) and disrupt the organization of MT minus ends at the spindle poles (Asteriti et al., 2011; Matov, 2024g). Further, elevated levels of the MT sequestering oncoprotein 18 (Op18) leads to the formation of small spindles (Houghtaling et al., 2009; Matov, 2024d) and has been associated with resistance to tubulin inhibitors (Cassimeris, 2002). Both TPX2 and Op18 do not directly affect MT polymerization turnover rates but regulate MT density and length, which – based on our new measurements – are the two parameters changed after overexpression of MYC.

Overall, the overexpression of MYC caused the formation of three types of abnormal spindles in RPE cells. Similarly to the rotation of the spindle in the most aggressive TNBC cells (MDA-MB-231), MYC overexpression induced a rocking motion after being constructively overexpressed (Vid. 8). In some spindles, MYC overexpression induced the formation of unfocused spindle poles – a hallmark of cancer (Vid. 9). Further, MYC overexpression induced the formation of multi-polar spindles (Therman and Timonen, 1950), also a well-characterized hallmark of cancer (Vid. 10).

Taken together, our new data indicates that MYC overexpression affects MT regulation in relation to the nucleation and branching of MTs but does not affect MT polymer turnover rates in dividing cells and therefore, targeting MYC overexpressing tumors with MT-stabilizing tumors, such as the taxanes, might be futile. It is our hypothesis that the continuous rotating and rocking motion of spindles overexpressing MYC results in improper chromosome segregation and, hence, aneuploidy, when the spindle assembly checkpoint (SAC) (Lara-Gonzalez et al., 2021) function is suppressed.

### Microtubule dynamics and drug resistance

#### Microtubule regulation and aneuploidy

In colorectal cancer cells, paclitaxel treatment could lead to overcoming chromosomal instability (CIN) and successfully progressing through mitosis (Ertych et al., 2014). CIN and impaired spindle checkpoint function are the result of reduced SAC mitotic arrest deficient 2 (Mad2) protein in *VHL*-defective cells (Thoma et al., 2009). Pathogenic levels of RIT1, a RAS-related GTPase, can suppress the SAC function by sequestering Mad2 from the mitotic checkpoint complex and promote chromosome segregation errors and aneuploidy (Cuevas-Navarro et al., 2021). RIT1 forms a signaling complex with Rac1 and enhances cell migration (Meyer Zum Büschenfelde et al., 2018). RIT1 has also emerged as an important GTPase in human pathogenesis (Van et al., 2020) and has been shown to drive oncogenic transformation in lung adenocarcinoma (Mozzarelli et al., 2025), suggesting targeting of RIT1. Oncogenic RIT1^M90I^ weakens the SAC and perturbs mitotic timing, resulting in sensitivity to Aurora A inhibition (Vichas et al., 2021). Aurora A regulates the MT minus ends at the spindle poles and its overexpression leads to SAC failure (Marumoto et al., 2005). Clinical trials are establishing the clinical utility of Aurora A inhibitors (Chakrabarti et al., 2024; Florou et al., 2024).

Differences in the ability of drugs to kill cancer cells stem from the level of drug-target engagement, which cannot necessarily be predicted by genetic sequencing alone. Paclitaxel reduces the tension at the kinetochores, but that is insufficient to induce Mad2 accumulation on kinetochores; instead, kinetochores unattached to MTs consistently bind Mad2 (Waters et al., 1998) and progress to anaphase. Drug treatments that lead transiently to mitotic arrest rather than cell death, which is the basis for drug resistance, require combination regimens (Verissimo et al., 2016).

Clinical data from RMC-6291 (Schulze et al., 2023), a KRAS^G12C^(ON)-only covalent inhibitor that depends on a tri-complex formation with CYPA (Cuevas-Navarro et al., 2025), has recently reported an objective response rate of 50% in patients with non-small cell lung cancer undergoing recent prior KRAS^G12C^(OFF)-only inhibitor treatment (Jänne et al., 2023). Further, early clinical data shows an approximately 38% unconfirmed response rate in patients with KRAS^G12X^ mutant lung cancer treated with the reversible multiselective inhibitor RMC-6236 (Arbour et al., 2023; Jiang et al., 2024). Acquired drug resistance limits the clinical impact of targeting RAS with TCIs that recruit CYPA to the active (ON) conformation of RAS. TCIs disable oncogenic signaling by preventing typical effector proteins, like RAF and PI3K, from binding to active RAS. Several clinical resistance alterations converge at attenuating the formation of the tri-complex, either by preventing drug-target engagement or by inducing RAF dimerization. One of the resistance mechanisms is acquired MEK1 mutations (Sang et al., 2025). The activity of MEK1 destabilizes interphase MTs and reduces mitotic spindle defects (Tolg et al., 2010). Furthermore, CYPA binds cytoplasmic dynein and co-localizes with MTs (Galigniana et al., 2004), suggesting the utilization of combination therapy that includes drugs modulating the activity (Garg and Alisaraie, 2025) of the MT minus end-directed motor dynein (Höing et al., 2018) to overcome drug resistance. Thus, quantitative live-cell microscopy analysis of MT dynamics can facilitate the optimization of lung cancer therapy.

We have shown that *VHL* mutations in the MT-interacting/HIF1α-interacting domain are more often linked to changes in MT polymerization and depolymerization rates (such as the *VHL* type 2A mutant pVHL30(Y98H)), while *VHL* mutations in the Elongin C-interacting domain are more often linked to changes in MT catastrophe and rescue frequencies (such as the *VHL* type 2B mutation pVHL30 (R161P)) (Thoma et al., 2010). Therefore, our hypothesis is that renal cell carcinoma tumors can be targeted with MT-stabilizing drugs. In *VHL* ^*−/−*^ cells, targeting the S-phase kinase associated protein 2 (Skp2) attenuates MT polymerization rates and leads to mitotic arrest and cell death (Pu et al., 2025), which indicates a clinical utility of Skp2 inhibitors, such as dioscin (Jiang et al., 2020). Further, aneuploidy-induced heterozygous VHL loss has been shown to stabilize HIF in lung cancer cells (Kopko et al., 2025). The clinical approval of HIFα inhibitors (Toledo et al., 2023) for the treatment of *VHL* deficient tumors indicates the importance of evaluating MT dynamics (Thoma et al., 2010) in patient-derived cells for such tumors to optimize therapy.

The variability in regulation and the redundancy in the activated pathways in disease demonstrate the need for quantitative live-cell microscopy methods that can determine the changes in morphology and dynamic organization within cells to predict clinical drug efficacy.

#### High dose docetaxel has less effects on microtubule dynamics

We have demonstrated that MT dynamics can predict resistance to tubulin inhibitors in pre-clinical data (Matov, 2024g). Tubulin inhibitors have been shown to be effective mainly during mitosis when used alone, but to induce apoptosis at all stages of the cell cycle – predominantly during G1 phase – when used in combination with other drugs (Mertens et al., 2023), which prompted us to further investigate interphase MTs.

**FIGURE 4:**
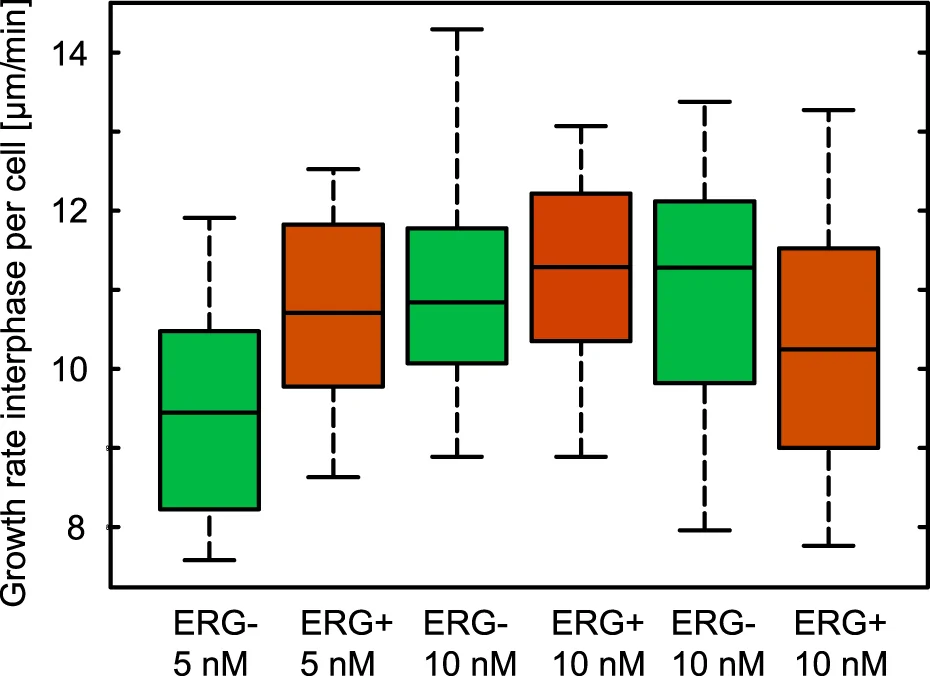
Effects of overexpression of ERG per cell. DU145 prostate cancer parental cell line and an ERG-overexpressing derivative cell line that shows a 20-fold increased docetaxel resistance were treated with 5 nM and 10 nM docetaxel. 5 nM docetaxel reduced MT polymerization rates in ERG-negative cells with 2.0 µm/min and increased them in ERG-positive cells with 1.1 µm/min. 10 nM docetaxel reduced MT polymerization rates in ERG-negative cells with 0.4 µm/min and increased them in ERG-positive cells with 1.6 µm/min. We repeated the 10 nM experiment (3rd and 4th boxplots) and measured that in ERG-negative cells docetaxel reduced MT polymerization with 0.5 µm/min and increased them in ERG-positive cells with 0.7 µm/min. The changes were not statistically significant. See Supplementary Table S3 for the number of videos and further statistics.

In prostate cancer (PC), chromosomal rearrangements often result in gene fusion of the *ERG* gene. The *TMPRSS2-ERG* fusion gene is the most frequent and is present in over 50% of patients (Tomlins et al., 2005). ERG overexpression is a negative prognostic factor for PC patients and confers docetaxel resistance. We have previously shown that 1 nM docetaxel attenuates MT polymerization rates with 3.3 μm/min in ERG-negative cells and increases them with 0.7 μm/min in an ERG-overexpressing derivative cell line that shows a 20-fold increased docetaxel resistance in terms of IC_50_ values (Galletti et al., 2014b). Here, we show new results that indicate that higher doses of docetaxel have less or no impact on the drug-sensitive cells. While the effects of 5 nM and 10 nM on the resistant cells remained the same (an increase in dynamics between 0.7 and 1.6 μm/min on average), the attenuation effect on sensitive cells decreased with the increase of docetaxel concentration. 5 nM docetaxel attenuated MT polymerization rates in ERG-negative cells partially with 2.0 μm/min, which is about 60% of the effect from 1 nM docetaxel. Finally, 10 nM docetaxel attenuated MT polymerization rates in ERG-negative cells with 0.4 μm/min and 0.5 μm/min in two different experiments (Figure 4; Supplementary Table S3). Taken together, our new analysis suggests that a low dose of docetaxel is more impactful.

**FIGURE 5:**
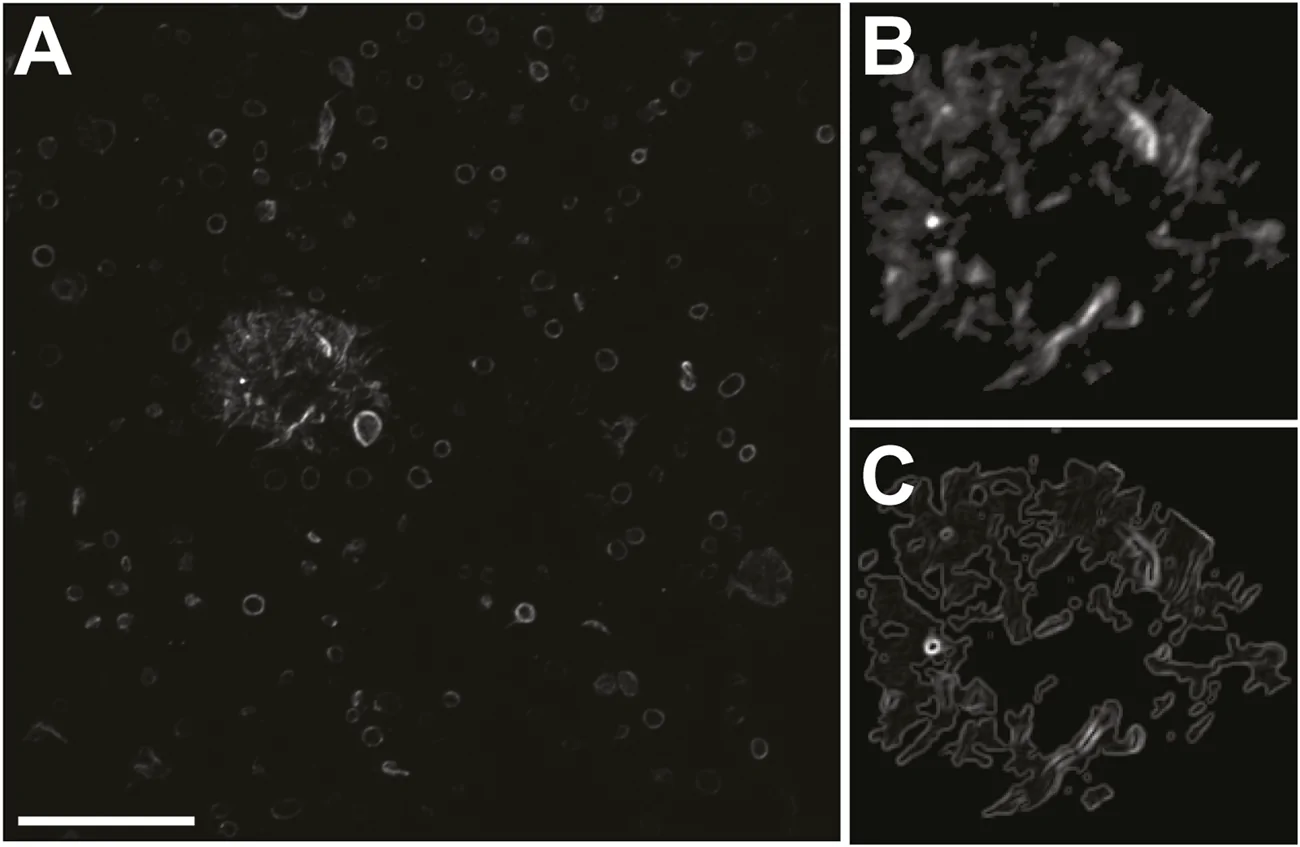
CTC of a metastatic castrate-resistant prostate cancer patient. Docetaxel-resistant patient CTC as well as the surrounding leukocytes, most of which are neutrophils. Besides such large cells, some CTCs with an aggressive phenotype are as small as lymphocytes (Matov, 2024d). Magnification 63×. Scale bar equals 20 µm. **(A)** Raw image of a CTC isolated from peripheral blood of a patient with stained α-tubulin. **(B)** Pixel intensity thresholding image. **(C)** Edge detection image of **(B)**.

Besides a MT tip dynamics assay, we have shown that image texture analysis (Haralick, 1979; Haralick, 1983; Haralick et al., 1973) could provide a quantitative readout of sensitivity to docetaxel by virtue of measuring MT cellular organization (Matov, 2024b; Matov, 2024i; Murphy et al., 2003). Evaluating drug-induced MT bundling is one possible approach to evaluating docetaxel activity. Specifically, we measure the effects of docetaxel on the spatial organization of the MT cytoskeleton. At baseline, the MTs are organized in a fine radial network (Figure 5) and docetaxel treatment in sensitive cells leads to a reorganization of the MTs into bundles of parallel aggregates.

In our analysis, the MTs are detected as above threshold intensity pixels and streaks of intensity gradients (Li et al., 2012; Matov, 2024b; Matov, 2024i; Shariff et al., 2010; Shariff et al., 2011). We estimate the angular orientation of the detected edges and bundle morphology, among other metrics. Importantly, the feature edge and morphology distinctions between cells are not always trivial to observe by naked eye and therefore computational methods (Murphy, 2011) are required to uncover a sensitivity-revealing organization and to distinguish it from the different patterns indicating intrinsic and acquired drug resistance (Matov, 2024b; Matov, 2024i).

Many mechanistic models have been proposed to describe drug resistance to tubulin inhibitors (Orr et al., 2003), the most common of which are: (i) resistance due to membrane-pump overexpression (Robert, 1999), (ii) resistance due to tubulin-isotype composition (Haber et al., 1995; Kanakkanthara et al., 2011; Ranganathan et al., 1998), and (iii) resistance due to mutations in drug binding sites (Giannakakou et al., 2000; Giannakakou et al., 1997), as well as (iv) resistance due to changes in regulatory microRNAs (Kanakkanthara and Miller, 2012). Evidence for these models stems largely from work with tissue culture cancer cell lines. Because these lines fail to adequately represent the genetic diversity found in the patient population, it is unclear how these findings relate to resistance to tubulin inhibitors in real tumors from patients. To begin translating our computational approaches to the clinic, next we investigated patient circulating tumor cells (CTCs).

### Analysis of circulating tumor cells

About a third of the PC patients progressing after treatment with hormonal therapies have intrinsic resistance to systemic therapy (Petrylak et al., 2004; Sweeney et al., 2015; Tannock et al., 2004; Vale et al., 2016). The two-thirds who respond, suffer visible side effects from drug toxicity and rapidly acquire resistance to therapy (Francini et al., 2019). Overall, the treatment of advanced PC is hampered by the lack of known molecular markers for reliable targeted therapies (Gao et al., 2014; Matov, 2025a).

Assays to detect CTCs in the peripheral blood of cancer patients have been used clinically to provide prognostic information and to test for minimal residual disease. Typical methods to detect CTCs include the use of monoclonal antibodies to detect tumor cells in the peripheral blood by immunocytochemistry or flow cytometry, or through the use of reverse transcriptase polymerase chain reactions (RT-PCR) to detect tumor cell-specific mRNAs. Each has its specific advantages and limitations. For example, RT-PCR is very sensitive and highly specific when the expression of the target mRNA is limited to malignant tumor cells. Flow cytometry, on the other hand, has been used to detect and authenticate cells such as CTCs, but it does not allow visual confirmation of cell morphology or discrimination of changes at the subcellular level (Racila et al., 1998). The challenges in CTC detection relate to the need for high sensitivity combined with high specificity. The epithelial cell adhesion molecule EpCAM, a transmembrane glycoprotein that consists of two epidermal growth factor-like extracellular domains, is detected at the basolateral membrane of the majority of epithelial tissues and is overexpressed by the majority of human epithelial carcinomas. Thus, use of antibodies to EpCAM (Momburg et al., 1987) allows for circulating epithelial-derived tumor cells to be isolated and concentrated from peripheral blood so they can be further inspected by microscopy. However, as EpCAM can also be expressed in leukocytes, the specificity of this antibody-based assay to detect CTCs is lowered to 80%. To increase the specificity of detection for epithelial tumor-derived cells, the CellSearch (Allard et al., 2004) technology uses three additional immunostains – an antibody against CD45 conjugated with allophycocyanin to identify white blood cells, a nuclear dye DAPI to fluorescently label the cell nuclei, and two phycoerythrin-conjugated anti-CK antibodies recognizing cytokeratins to more specifically identify epithelial cells as such. Stained cells are analyzed on a fluorescence microscope using the Cell Track Analyzer II. Automatically selected images are then reviewed by a human observer for manual inspection and counting of CTCs. The criteria for an object to be defined as a CTC include (i) round to oval morphology, (ii) a visible nucleus (DAPI staining), (iii) positive staining for CKs, and (iv) being negative for CD45 expression (CK8+/CK18+/CK19+/DAPI+/CD45-). In addition, the consensus pathognomonic features of epithelial tumor cells, such as a clearly enlarged nucleus, high nucleus-to-cytoplasmic ratio and/or clusters of two or more immunopositive cells are also part of the CTC definition criteria developed in the CellSearch method to avoid false positive selections. The main concerns regarding its accuracy, however, are in regard to its sensitivity – as up to 75% false negative (FN) detections have been reported (personal communications). To remedy this shortcoming, we worked on the development of an approach for CTC analysis in which no enrichment is used (Matov, 2024h; Tasaki et al., 2014), similarly to the “no cell left behind” approach (Cho and al., 2012), as well as a new enrichment method (Sung et al., 2013; Sung et al., 2012).

Our analysis includes not only CTC enumeration, but also a numerical evaluation of a variety of CTC morphology and texture metrics, which are related to the cellular localization and organization of proteins of interest that serve as biomarkers of disease progression and drug resistance in metastatic cancer (Matov, 2024h). Changes in the number of CTCs serve as a prognostic and predictive biomarker in the management of metastatic disease (Sung et al., 2013; Sung et al., 2012; Tasaki et al., 2014). The TAXYNERGY trial evaluated clinical benefits from early taxane switch – from docetaxel to cabazitaxel – and CTC biomarkers to interrogate mechanisms of sensitivity or resistance to taxanes in men with chemotherapy-naïve metastatic castrate-resistant PC (mCRPC) (Antonarakis et al., 2017; Sung et al., 2013; Sung et al., 2012). Our work has demonstrated that MT tips can be labeled and computationally analyzed in organoids (Matov, 2025a) derived from cancer patient CTCs (Gao et al., 2014).

Changes in CTC morphology and subcellular localization of proteins of interest can also be utilized to evaluate disease progression and drug efficacy. We have developed methods for both high-resolution and low-resolution detection and analysis of CTCs in patient peripheral blood samples (Matov, 2024h) as well as methods for 3D analysis of biomarkers in image stacks (Matov, 2024b). Our analysis is based on the detection of multiple (up to five) fluorescent labels in multiplex microscopy images. Staining for CTCs, termed a positive cell identifier, is done with either PSMA or CK labeling. Cell nucleus staining is done with DAPI labeling. Staining for leukocytes, termed a negative cell identifier, is done with CD45 labeling. This way, a CTC is most commonly a cell that is PSMA+/DAPI+/CD45-, that is, it is an epithelial cell expressing PSMA, it has a nucleus, and it is not a blood cell. Additional markers we analyze are α-tubulin and the androgen receptor (AR). MT reorganization and AR nuclear localization are considered readouts of drug efficacy (Matov, 2024d; Thadani-Mulero et al., 2012; Thadani-Mulero et al., 2014).

Figure 5 shows a high-resolution CTC of a docetaxel-resistant patient with basic image processing manipulations demonstrating the heterogeneity of the edges of the intricate network of the MT cytoskeleton area of the cell. Texture and morphology analysis allows to detect response to treatment, or a lack thereof (Galletti et al., 2013; Galletti et al., 2014a). We have shown that treatment with docetaxel causes CTCs to become smaller (Matov, 2024i), which indicates resistance to tubulin inhibitors. Treatment with androgen inhibitors is also associated with the emergence of a very aggressive phenotype of CTCs that are as small as lymphocytes (Matov, 2024d). Such cells have circumvented the need for androgen (Bander et al., 2005), which affects their metabolic and cell growth patterns. Advanced prostate tumors often bypass the functional requirement for AR through epigenetic cellular plasticity, evidenced by the manifestation of neuroendocrine transdifferentiation (Davies et al., 2018). FGF signaling can bypass a requirement for AR, and FGF and MAPK pathways have been shown to be active in metastatic AR-null PC (Bluemn et al., 2017).

This variety in regulation and, thus, the morphology of the CTCs in advanced disease highlights the importance of the utilization of advanced computer vision methodology in order to reliably enumerate CTCs and evaluate quantitatively all available cellular biomarkers.

**FIGURE 6:**
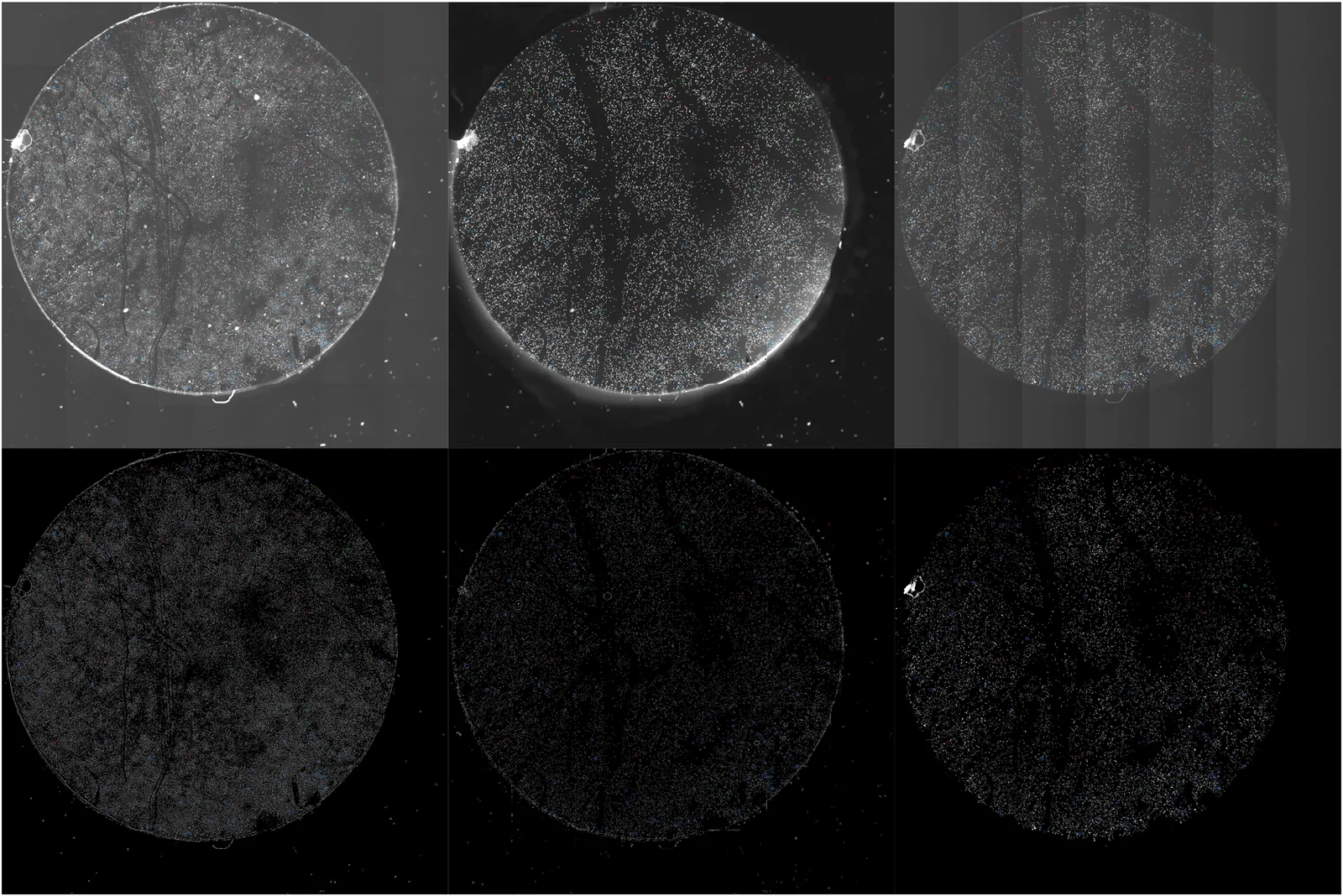
PSMA/DAPI/CD45 labeling in an 8 mm coverslip of peripheral blood. Metastatic patient CTCs are detected as PSMA+/DAPI+/CD45- blobs. The images are composite and consist of 49 tile images (7x7) per coverslip. A coverslip has a diameter of 8 mm. Magnification 10×. Upper left panel is a PSMA image. Upper middle panel is a DAPI image. Upper right panel is a CD45 image. Lower left panel is PSMA segmentation. Lower middle panel is DAPI segmentation. Lower right panel is CD45 segmentation. Segmentation is done with a stationary wavelet transform (Olivo-Marin, 2002) and unimodal pixel intensity thresholding (Rosin, 2001). Green squares mark the CTCs (TP). Blue squares mark the leukocytes (TN). Red squares mark the FP. The FP selections are subjective, for these cells there is a disagreement between the human eye and the computer vision, and were later reclassified as TP after further manual inspections. There were no FN selections. Because of the large image size (the original image is 133 MB and a compressed JPG copy is 24 MB), it is difficult to see the labels and a compressed image is uploaded for viewing also at: https://github.com/amatov/SegmentationBiomarkerCTC/blob/main/Coverslip/p001_1waveletResized.jpg.

In our experience, low-resolution imaging at 10× magnification allows for automated enumeration of CTCs in peripheral blood. Figure 6 shows multiplex microscopy images of an 8 mm coverslip. The three channels imaged are PSMA, DAPI, and CD45 (Sung et al., 2013; Sung et al., 2012; Tasaki et al., 2014). We detected 23 CTCs, which was confirmed by expert manual enumeration, and the algorithm did not miss any CTCs. In addition, we detected 16 cells classified as false positive (FP), because even if our algorithm detected them, they were not listed in the coordinate list with manual selections. Our results indicate that, because it is very difficult to reliably see with naked eye low pixel intensity values, in particular when they are adjacent to bright image areas (Aubert, 1865), it seems that the computer algorithm might be able to perform better in such scenarios than the human observer. A secondary manual inspection of the 16 cells selected by our computational algorithm confirmed them as true positive (TP) selections. In some cases, we had disagreements between different human observers and had to manually examine the different imaging channels at high magnification. We also performed image contrast enhancement steps to highlight particular features of the detected cells to facilitate the determination. In our experience, the variability in the brightness of the PSMA staining is the main obstacle to reliable detection of CTCs by a human observer. We apply computational models of the fluorescent signals and reconstruct the nature of the underlying processes. Digital image analysis serves as an indispensable tool for the interpretation of the lost during signal formation information. Furthermore, computer vision analyses deliver incredibly fast processing of imaging data in comparison to humans.

**FIGURE 7:**
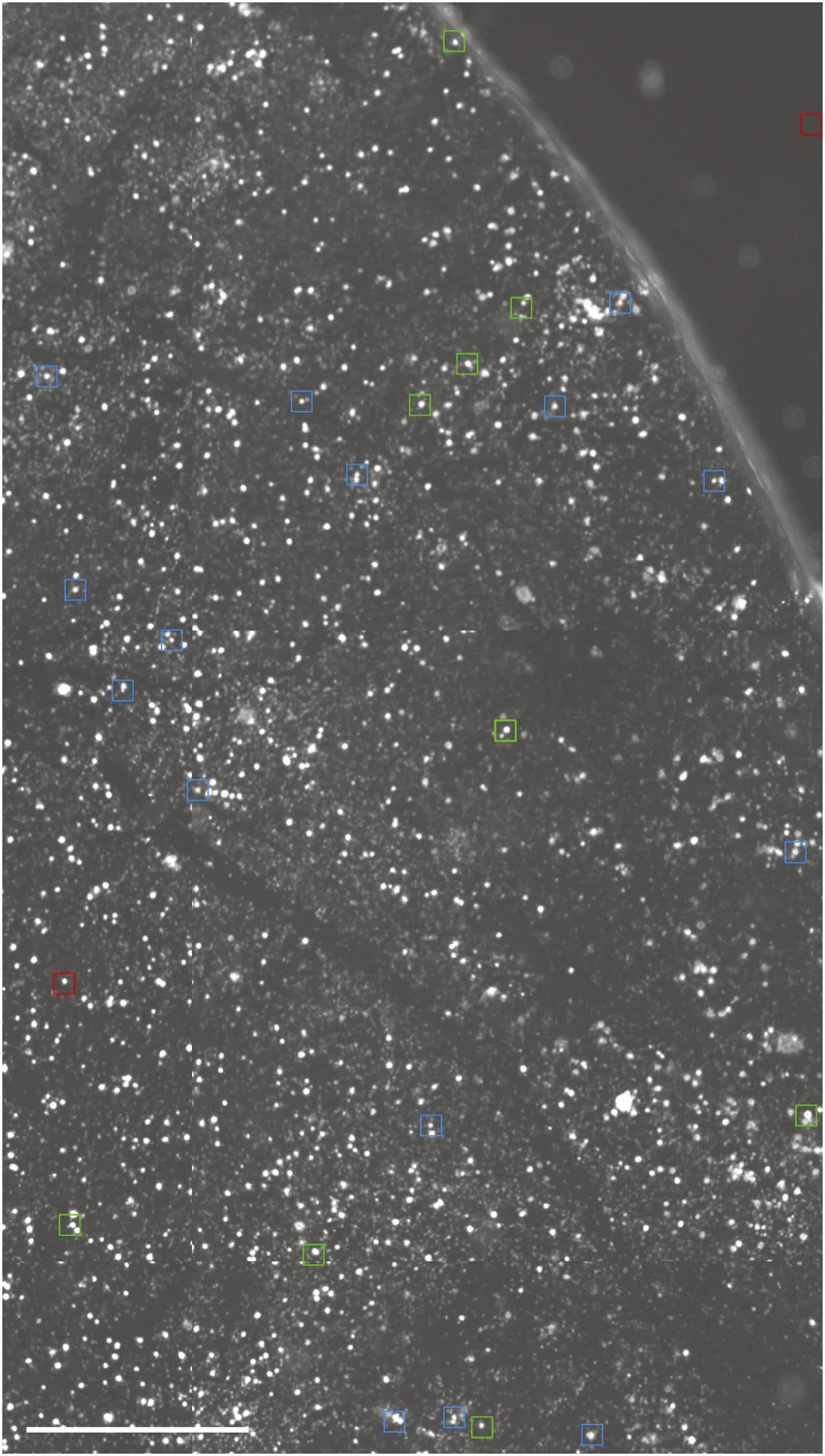
PSMA labeling in an 8 mm coverslip of peripheral blood. Metastatic patient CTCs are detected as PSMA+/DAPI+/CD45- blobs. Magnification 10×. Scale bar equals 450 nm. The figure shows the same coverslip and detections as in Figure 6, but only a small portion of the upper-right corner of the PSMA channel is shown with overlaid detections. Green squares mark the CTCs (TP). Blue squares mark the leukocytes (TN). Red squares mark the FP. Note the FP in the upper-right corner of the image, resulting from the border effect between adjacent tiles. A larger size version of this image can be viewed at: https://github.com/amatov/SegmentationBiomarkerCTC/blob/main/Coverslip/p001_1crop.jpg.

In summary, the benchmark result is 39 (of 39) TP / 0 FP / 0 FN. We also detected a very large number of leukocytes (PSMA-/DAPI+/CD45+), which are the true negative (TN) selections. Figure 7 shows a few tiles of the composite image from Figure 6, namely, an area in the upper-right corner of the PSMA channel. The green squares show the TP (that is, the CTCs), the two red squares show the FP, and the blue squares are the TN (the leukocytes). Observe in the upper-right corner of Figure 7 the FP selection (in red) made at the edge of the tile, as border areas can be problematic when there is an intensity mismatch between neighboring tiles. During software calibration – setting the thresholds of the ranges of cell size and number of CTCs accepted by the automated algorithm – and validation, we were guided also by available clinical data (Armstrong et al., 2012) and ERG rearrangement status (Mosquera et al., 2007).

Our software allowed us to reduce the time for analysis per 8 mm coverslip to 5 min, which was significantly less than the 4 h required for a manual inspection and detection of CTCs. The prolonged and tedious manual selection process is proned to missing CTCs and when such cells are discovered during a second examination of the same coverslip, it triggers a third and perhaps a fourth round of manual inspection, which adds many hours of additional validation. Our eyes and visual perception are incapable of reliably and reproducibly analyzing image patterns when the contrast is low. For the computer program, the image is a collection of pixels, each of them with an intensity value, and the readout from a particular pixel is not influenced by the brightness of the surrounding pixels (or the level of lighting in the room).

In addition, we analyzed tyrosinated tubulin (Dunn et al., 2008), which appeared as bright MT rings (Matov, 2024d) in low-magnification images – and AR nuclear localization (Thadani-Mulero et al., 2012) in 3D datasets (Matov, 2024b), and the patterns in these two channels were considered biomarkers of sensitivity to drug action. In drug-sensitive interphase cells, MT bundle formation is the result of drug-mediated MT polymer stabilization and as such it, represents a hallmark of taxane cellular activity (Sung and Giannakakou, 2014). MT bundles eventually form circular shapes as cells approach apoptosis. We applied a circular Hough transform to detect MT bundling and reorganization in drug-treated cells (Matov, 2024d). Hough transform is a computational technique, which allows the automated detection of a variety of shapes in images (Hough, 1959), most commonly used in the area of computer vision for line detection (Mattavelli et al., 2001). It transforms image space into parameter space, enabling the detection of shapes through pattern identification in the transformed space. We validated our computational approach by comparing MT bundling in paclitaxel-treated drug-sensitive and drug-resistant cancer cells (Matov, 2024d). Our measurements indicated that 83% of the drug-sensitive cells form MT bundles after drug treatment, 9.7% are affected by the drug, but do not form bundles, and 7.3% appear apoptotic. In contrast, only 27% of the drug-resistant cells respond by forming MT bundles, 58% do not form bundles at all, 7.5% are partially affected, but do not form clear bundles, and 7.5% are apoptotic. Further, when we compared the pixel intensity of the bundled MTs in the affected cells, the overall brightness was 70% higher in the paclitaxel-susceptible cancer cells (Matov, 2024d). These results indicate that even if there are measurable drug-induced changes in about a quarter (27%) of the drug-resistant cells, the effects of paclitaxel are significantly reduced and there are fewer MTs within the bundles. Overall, these data demonstrate two different mechanisms of taxane resistance.

AR subcellular localization can be utilized to evaluate disease progression and drug efficacy since its drug-induced translocation to the nucleus is considered a response to therapy (Thadani-Mulero et al., 2014). We quantified the percentage of nuclear AR of the volume (based on 3D image stacks) of the cells, as readouts of effective drug-target engagement in patient-derived CTCs (Matov, 2024b). Of the 10 coverslips with patient samples we analyzed, half were sensitive and half were resistant to clinical treatment with docetaxel. Our analysis indicates that drug-sensitive patients have an average of 52% of nuclear accumulation of AR, while in drug-resistant patients it is reduced to an average of 24%. Our measurements further indicate that while AR translocation to the nucleus can be indicative of susceptibility to drug action, there is a high level of variability (43%-70% nuclear AR) among the patients who respond, while in the about a quarter of the patients with refractory disease the numbers are more uniform – 15%-31% of the AR is in the nucleus when the patients are resistant (Matov, 2024b). This result suggests that when patients respond to therapy, the response is partial.

The main difficulty we faced analyzing such low-resolution image datasets was the presence of double positive as well as double negative cells, that is, cells with both PSMA and CD45 labeling or with none. To provide an example from the analysis of CTCs collected with a geometrically enhanced differential immunocapture (GEDI) microfluidic device (Sung et al., 2013; Sung et al., 2012), our computational analysis identified 297 CTCs. Within these cells, which we detected in one-eighth of the overall GEDI chip surface, manual validation determined that 230 were CTCs, eight were leukocytes (2.7% FP), 28 were double positive cells, and 31 were double negative cells. In our view, because the PSMA staining is intrinsically less bright than the CD45 staining, when the two seem to a human observer to be similar (that is, double positive or double negative staining), the cells are CTCs – in particular, because the automated algorithm has measured higher values in the PSMA channel – and therefore, the benchmarking indicates 97.3% TP selections. The wrong classification of a few leukocytes can be corrected with improved geometric considerations.

**FIGURE 8:**
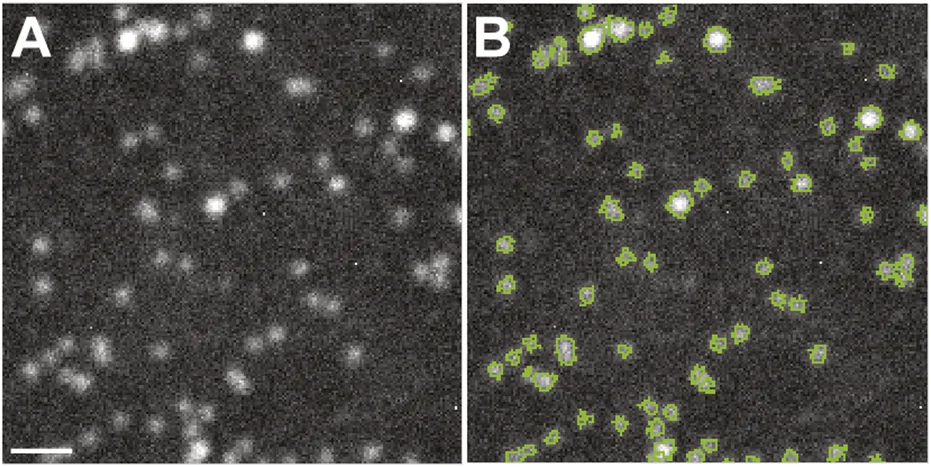
Expression levels of PSMA in patient cells collected from a peripheral blood draw of a metastatic castrate-resistant prostate cancer patient. **(A)** Raw image of PSMA labeling in CTCs. Magnification 63×. Scale bar equals 70 µm. **(B)** PSMA segmentation based on stationary wavelet transform, active contour, and watershed transformation (Matov, 2024h). The edges of the segmented PSMA areas are displayed in green.

Figure 8 shows the segmentation of the precise contour of the PSMA label in a high-resolution image at 63× magnification, which allows for a more detailed characterization and evaluation of the resistance biomarkers. Our measurements indicated that docetaxel treatment affects multiple numerical parameters (Boland and Murphy, 2001) describing the changes in morphology and texture of the MT network leading to, for instance, bundling, modulation of cell shape, surface patterns, and size (Matov, 2024g). To translate our analysis of fixed patient cells to living patient cells, next we interrogated the dynamics of cellular organization in patient-derived cancer organoids (Matov, 2025a; Matov et al., 2016).

PSMA, a cell surface antigen overexpressed in PC, provides a validated target (Bander et al., 2005). ^**225**^Ac-J591, anti-PSMA monoclonal antibody J591 radiolabeled with the alpha emitter actinium-225 is being investigated in the context of safety, efficacy, maximum tolerated dose, and recommended for phase II dose (Tagawa et al., 2024). PSMA expression is found in higher grade PC as well as mCRPC (Bostwick et al., 1998), while its expression is low in normal tissues (Silver et al., 1997), and recent clinical trials have validated PSMA as a target for therapy for high-risk and advanced PC (Sartor et al., 2021), demonstrating it may offer benefits over the use of tubulin inhibitors (Hofman et al., 2021). PSMA targeting led to PSA decline in 95% of the patents and all patients had at least 50% decline in CTC count or converted to undetectable levels of CTCs after 12 weeks of treatment. Patients experienced minimal adverse effects and did not suffer immediate or significant salivary gland toxicity as well as the potential delayed renal toxicity (Nauseef et al., 2023).

### Analysis of patient-derived cancer organoids

The treatment of advanced PC is hampered by the lack of known molecular markers for reliable targeted therapies (Network, 2015). In this context, PSMA offers a much-needed target and clinical trials (recommended for phase II dose (Tagawa et al., 2024)) are demonstrating that patients experience low levels of toxicity and manageable side effects.

**FIGURE 9:**
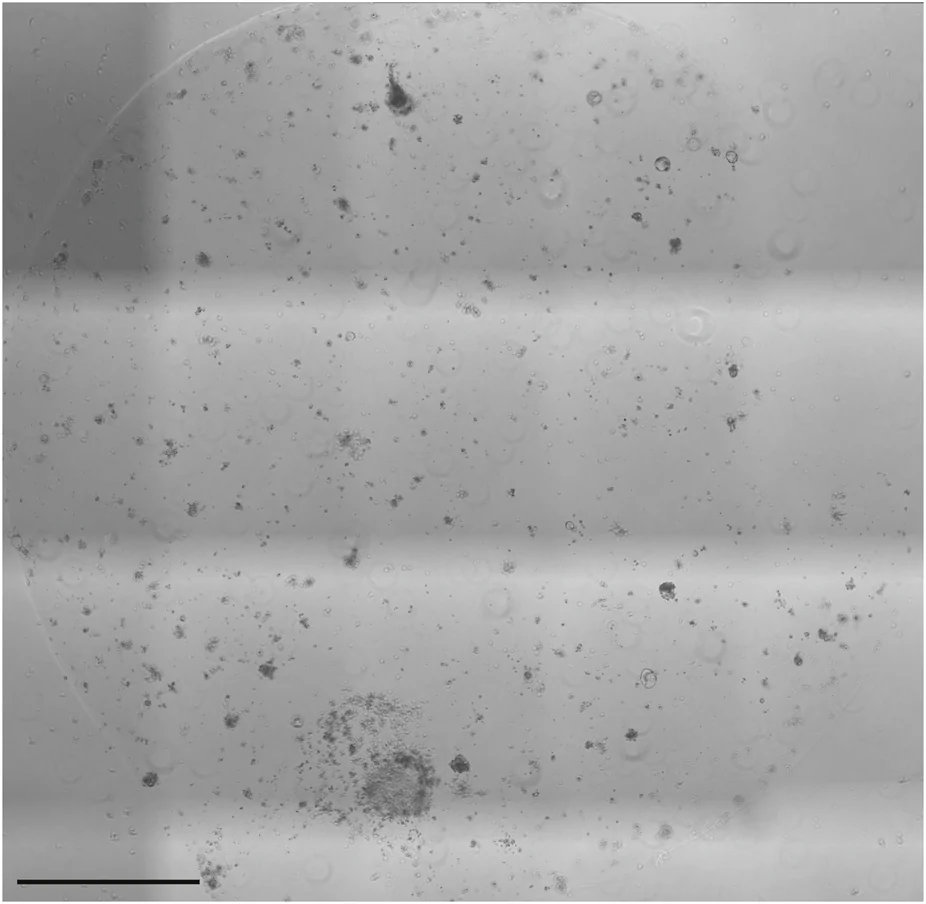
Retroperitoneal lymph node metastasis patient-derived prostate cancer organoids after treatment with 1 nM cabazitaxel. Image shows a 30 µL Matrigel drop. Transmitted light microscopy. Magnification 4×. The diameter of the drop is 60 mm. Scale bar equals 11 mm. The image is composite and consists of 20 tile images (4x5). Because of the large image size (the original image is 45 MB), it is difficult to see the receding cancer organoids and a compressed 16 MB image with cropped lower five tiles is uploaded for viewing also at: https://github.com/amatov/TextureFlowAnalysis/blob/master/Large%20Image_1nanoCBZ_01crop.tif.

To anticipate drug resistance and elucidate mechanisms of resistance, we derived primary tumor and metastatic organoids from resected tissues from PC and colorectal cancer patients, and treated them with docetaxel and cabazitaxel. Figure 9 shows a Matrigel drop of about 60 mm in diameter with metastatic PC organoids treated with 1 nM cabazitaxel for 21 days. In the lower part of the image is visible a cluster of dead cells from a very large organoid. Besides texture and morphology analysis of organoid surface (Matov, 2024h), we have proposed methodology for the analysis of subcellular dynamics in such datasets (Matov, 2024i). Automated quantitative imaging approaches for disease management can be a useful addition to the currently used methodology in the clinic (Petrylak et al., 2004).

Our quantitative live-cell image analysis indicated that ixabepilone rather than the taxanes affects MT regulation in receptor-negative and HER2-positive BC cells. Conversely, ixabepilone was less impactful compared to the taxanes in ER-positive cells (Matov, 2024f) and its activity in ER-positive cells can be increased by the depletion of βIII-tubulin (Lopus et al., 2015). MT lattice binding of CLIP170, which preferentially binds (Perez et al., 1999) and tracks (Thoma et al., 2010) MT ends, is another mechanism of resistance to MT-stabilizing drugs (Thakkar et al., 2021). Mechanisms of resistance to tubulin inhibitors can further be elucidated and validated by the computational analysis of patient-derived cells cultured as organoids.

Patient-derived organoids (Sachs and Clevers, 2014) can be labeled with fluorescent markers of key cellular proteins that deliver a detailed insight into the metabolic changes in patient tumor cells after drug treatment. In a primary colon cancer organoid (van de Wetering et al., 2015), the dynamics of EB1 comets indicate a visible deterioration of MT dynamicity after treatment with 2 nM vinorelbine (Vid. 11). We have proposed a computational approach for the selection of tubulin inhibitors in solid tumor oncology based on the analysis of MT dynamics in patient cells (Matov, 2024d; Matov, 2024f; Matov, 2024g; Matov, 2025a). An important component to optimizing a drug regimen is also refining drug dosage as there are non-linear pharmacodynamics (Potashnikova et al., 2019) associated with treatment with tubulin inhibitors (Matov, 2024b; Matov, 2024i).

It is our hypothesis that some of the current drug regimens are administered in ways that might benefit tumor survival and in other circumstances the drugs could be failing to target the presence of, induced by previous treatments, a vulnerable tumor cell state. Approximately 30% of receptor TNBCs exhibit functional loss of the retinoblastoma protein tumor suppressor suggesting a vulnerability (Matov, 2025a) and susceptibility to PLK1 inhibition (Witkiewicz et al., 2018). In such tumors, treatment with paclitaxel (or other taxanes) could – counterintuitively – help the cancer cells progress through mitosis (Shannon et al., 2005) by dampening MT dynamics during mitosis, and thus reducing chromosomal instability in dividing tumor cells (Ertych et al., 2014).

Many of the drug-target interactions occurring in patient tumors cannot be evaluated by genetic sequencing alone and require integrating quantitative microscopic evaluation of patient-derived tumor cells into the process of making clinical decisions. We consider this particularly important in regard to the choice of a drug regimen in advanced tumors that are (currently) considered incurable (Bardia et al., 2021). The reason is that there are examples in oncology for complete and long-lasting remission of very advanced tumors for which approved treatment options have been exhausted (Linn et al., 1994). We hypothesize that we can (i) identify an ultimate responder per each molecular subtype (that is, a regimen resulting in a complete response) and (ii) sensitize every tumor to one of the ultimate response regimens by modulating cytoskeletal dynamics and cellular organization.

### Acetylation and GSK3β targeting

We have shown that tubulin acetylation (or the loss of pVHL) modulates MT dynamics by reducing the frequency of MT catastrophe and that it protects MTs from the activity of depolymerizing agents, for instance, nocodazole (Matov et al., 2010; Thoma et al., 2010). It is conceivable that acetylation reduces significantly the rates of catastrophe of astral MTs in rotating MDA-MB-231 and HBL-100 spindles, and the spindles continue rotating without being able to properly align the chromosomes before proceeding to anaphase. We showed that treatment with a HDAC6 inhibitor sensitized MTs to the effects of nocodazole by inhibiting tubulin acetylation (Matov et al., 2010) and such treatment might sensitize TNBC tumors to other MT-destabilizing drugs, such as vinca alkaloids or eribulin (Smith et al., 2010).

**FIGURE 10:**
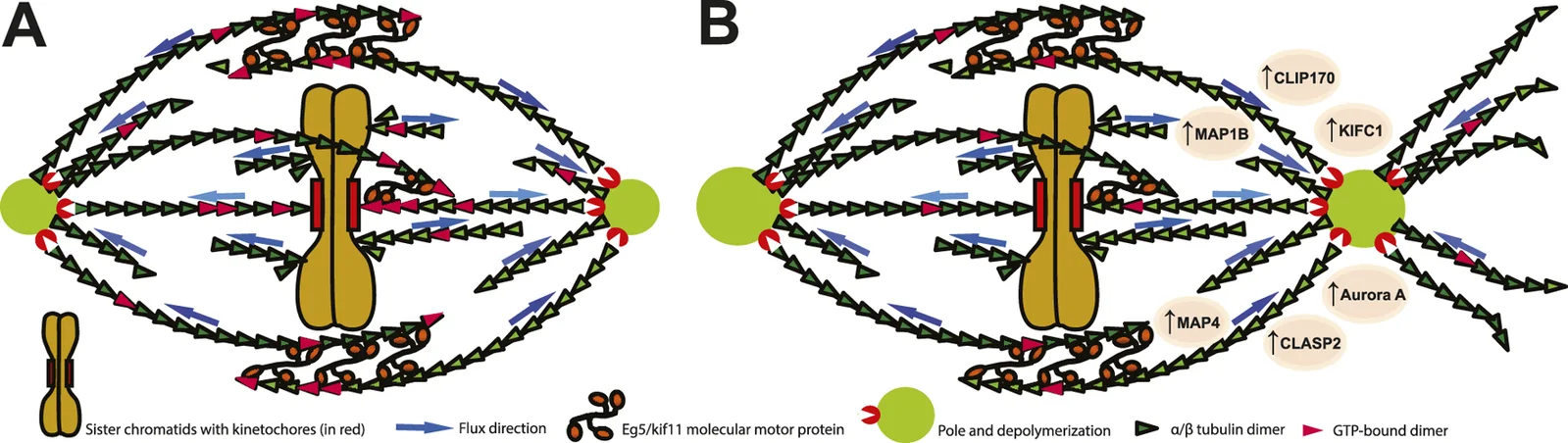
Dynamic organization of the mitotic spindle. During metaphase the sister chromatids are aligned at the metaphase plate by MT bundles attached to the kinetochores. Opposite interpolar MT bundles overlap and are interconnected via plus-end directed molecular motor proteins (Vale and Milligan, 2000). MTs have their minus ends toward the spindle poles, where they remove dimers, that is, depolymerize. MTs have their plus ends toward the metaphase plate, where they switch between states of polymerization and depolymerization, a process known as “dynamic instability”. Panel A depicts a cell in normal physiology for which some GTP remnants are present along the MT lattice; they are often the site of MT rescue, that is, the sites where MTs switch to a phase of polymerization. Panel B depicts a cancer cell in which fewer MTs are GTP-capped and there are less GTP remnants along the MT lattice; the spindle poles are unfocused and occupy a larger area. In such cells, the MTs are more likely to switch to depolymerization and less likely to pause or switch to polymerization (Matov, 2024g; Thoma et al., 2010), which would result in segregation errors and improper chromosome segregation. Proteins in the MT transcriptome, like Aurora A, CLASP2, CLIP170, MAP1B, MAP4, and KIFC1 are upregulated in cancer cells. These proteins are involved in the regulation of the minus ends of MTs and their targeting can sensitize tumors to systemic therapy.

Targeting MAPs that promote rescue (Howard, 2001), such as CLIP170 (Hu et al., 2022) and CLASP2 (Luo et al., 2023), and their downregulation (Figure 10) might be another avenue to sensitize TNBC to the action of tubulin and/or acetylation inhibitors. Further, MAP1B is overexpressed in TNBC, but not in other BC molecular subtypes, and is a prognostic and therapeutic target (Inoue et al., 2024). MAP4 is upregulated in TNBC as well (Wang et al., 2022). GSK3 inhibitors are currently being tested for over 20 clinical indications from neurodegeneration to a variety of cancers (Duda et al., 2020; Wood, 2022). MAP1B and MAP4 (Figure 10), which are downstream of GSK3β, bind the MT lattice and suppresses polymer turnover, and could be targeted by inhibiting GSK3β (Kumar et al., 2009) to prevent the formation of long astral MTs in combination with an acetylation inhibitor.

GSK3β and pVHL cooperatively maintain primary cilia (Davenport and Yoder, 2005) in renal cell carcinoma cells. pVHL plays a key role in maintaining the structural integrity of the primary cilium, a MT-based cellular antenna important for suppression of uncontrolled proliferation of kidney epithelial cells and cyst formation. The cilium maintenance function of pVHL is independent of its role in HIF1α degradation, but it is dependent on its ability to stabilize the MT network of the ciliary axoneme (Thoma et al., 2007a). pVHL-negative cells lose their primary cilia in response to serum stimulation. The effect of serum on pVHL-negative cells is dependent on PI3K signaling and could be mimicked by inhibition of GSK3β. Thus, pVHL and GSK3β demonstrate a genetic redundancy with respect to cilia maintenance. Inhibition or loss of either protein alone has no effect on the primary cilium, but inhibition or loss of both causes cells to rapidly lose their cilia (Thoma et al., 2007b). We have shown (Matov, 2024d) that growth factor deprivation enhances MT polymerization turnover and destabilizes MT, and that GSK3β-mediated phosphorylation and inhibition of pVHL function induce these changes in MT homeostasis.

Phosphorylation of pVHL at Ser-68 proceeds mainly in serum-starved cells and is consistent with the fact that GSK3β is predominantly active under conditions of serum starvation (Lochhead et al., 2006). Our measurements of EB3 comet speeds after perturbations of the GSK3β-pVHL axis indicated altered MT dynamic instability. Specifically, serum-starvation of pVHL-positive retinal pigment epithelial cells altered MT polymer turnover to the same extent as shRNA-mediated depletion of pVHL in exponentially-growing cells when serum was present (Matov, 2024d). Importantly, inhibition of GSK3β in serum-deprived cells attenuated MT polymer turnover, which reached rates similar to those we measured in exponentially-growing pVHL-positive cells. To achieve GSK3 inhibition, 20-h starvation was followed by a 4-h incubation with 100 μM GSK3 inhibitor VIII (AR-A014418), which is used in the treatment of PC (Li B. et al., 2015). Our measurements suggest that the state of quiescence is characterized by increased MT dynamics, as compared to the state of exponential growth, and that GSK3β plays a critical role in mediating the changes in MT dynamics (Matov, 2024d). To assess whether GSK3β-mediated phosphorylation of pVHL contributes to the measured changes in MT dynamics associated with quiescence, we analyzed serum-starved renal cell carcinoma cells producing either the phosphorylation site mutant pVHL30(S68/72A) that is resistant to phosphorylation by GSK3β or the phosphorylation-mimicking mutant pVHL30(S68/72D) (Matov, 2024d). Serum deprivation caused an increase in MT turnover in renal cell carcinoma cells that expressed pVHL30 or pVHL30(S68/72D), but not in cells expressing pVHL30(S68/72A).

Our analysis has indicated that the state of quiescence is characterized by increased MT dynamics, compared to the state of exponential growth, and that GSK3β plays a critical role in mediating the changes in MT dynamics (Matov, 2024d). The increase in MT dynamicity is similar to the changes caused by inactivation of pVHL (Thoma et al., 2010), which causes neoplastic transformation in renal cells. In this context, it is our hypothesis that in drug-naïve low-grade prostate tumors (while GSK3α is overexpressed and GSK3β is not (Duda et al., 2020)), a reverse differentiation could be induced by an inhibitor of GSK3 in combination with other compounds that collectively restore MT dynamics to their levels in benign prostate cells (Matov, 2025a). We further hypothesize that tumors of the prostate, of which only a small fraction were considered immunogenic (Shenderov et al., 2023; Wu et al., 2018), can be modulated with compounds that alter their cytoskeletal regulation to become susceptible to immunotherapy.

### Probing cytoskeletal organization in NK Cells

The upregulation of the six proteins shown on Figure 10 in cancer might predispose tumor cells to necroptosis, similarly to the effects of the upregulation of Rac1 and Rho in cell lines (Zhu et al., 2021). To elucidate the programmed cell death pathway activated by the immune system (Mitchison, 2021), patient tumor cells and lymphocytes, such as natural killer (NK) or T cells, can be co-cultured as organoids and their intercellular and intracellular dynamics analyzed via the methodology we propose.

In NK cells, lytic granules (cytotoxic lysosomes containing granzymes) are closely associated with MTs and F-actin (Mace and Orange, 2012). Quantitative imaging of the pore-forming protein (perforin) and lytic granules released in cancer cells, and trafficked by dynein on MTs toward the centrosome (the MT-organizing center) at their minus ends (Mentlik et al., 2010), could be utilized for evaluating the efficacy of cell therapy in real time (Matov, 2024i; Matov, 2024j). Talin association with another FA protein, vinculin (Adams et al., 2004; Matov, 2024d), recruits the Arp2/3 complex to initiate new actin filaments (Mullins et al., 1998) and, thus, integrin signaling (Brakebusch and Fassler, 2003) promotes F-actin reorganization for NK cell mechanotransduction (Wong and Ding, 2023). Probing the cytoskeletal organization of F-actin, MTs, and FAs in NK cells by a combination of quantitative imaging techniques (Matov, 2024e; Matov, 2024g; Matov, 2024i; Matov et al., 2005; Spanjaard et al., 2015) may allow us to anticipate which pathway is activated in a patient. Furthermore, it may equip us with a computational tool for the identification of small molecules that enhance the efficacy of NK cells and personalize treatment.

NK cells remain intact after releasing their cytotoxic content into the target cell because of lipid rafts that protect them from the action of perforin (Rudd-Schmidt et al., 2019). It is conceivable that some tumor cells are not affected by NK cells because of a similar mechanism. To sensitize such tumors to the effects of cytotoxic immune cells, lipid rafts can be disrupted by the depletion of cell membrane cholesterol in tumor cells. This can be achieved by the utilization of synthetic nanoparticles mimicking the cholesterol-rich high-density lipoproteins that bind scavenger receptor type B-1 and remove cholesterol through this receptor (Plebanek et al., 2015).

### Probing cytoskeletal organization in T cells

Further, we hypothesize that small molecules can alter the immunogenicity of tumor cells and promote tumor-specific T-cell activation in tumors currently considered non-immunogenic (Wu et al., 2018). This can be tested by isolating T cells from patient blood and co-culturing them with patient-derived cancer organoids. A tumor with a high number of tumor-infiltrating lymphocytes (TILs) could have hundreds or thousands of T cells and other immune cells that are all tumor-specific (Pardoll, 2012). In PC, primary tumors or peripheral blood would not contain a sufficient number of tumor-specific T cells (Sena et al., 2021), but it is feasible to isolate a small number of TILs from patient samples and expand these cells *ex vivo* (Dijkstra et al., 2018).

We derived a PC organoid from micro-metastasis at a retroperitoneal lymph node for which whole genome sequencing showed homozygous mutations in *PTEN* (exon2, c. G194>C, p. C65S) and *BRCA2* (exon14, c. T7397>C, p. V2466A) as well as heterozygous KIF4B (exon1, c. G1739>T, p. R580L) and Rap1GAP (exon8, c. G511>A, p. A171T) mutations. KIF4B is a protein, which is important in the spindle midzone (Bieling et al., 2010) and suppresses MT dynamic instability by dampening both polymerization and depolymerization rates at their plus ends (Yildiz, 2025). Knockout of KIF21B, another kinesin-4, causes MT overgrowth and perturbs centrosome and T-cell polarization. Functioning KIF21B induces catastrophe, restricts MT length, and permits centrosome translocation toward the target cells by dynein-mediated pulling forces that allows the centrosome to localize past the nucleus (Hooikaas et al., 2020).

EB1 interacts with T-cell receptor cytosolic regions and mediates the organization of an immunological synapse to transduce activation signals (Lasserre and Alcover, 2012). Lytic granules from cytotoxic T cells have been shown to exhibit kinesin-dependent motility on MTs (Burkhardt et al., 1993). T-cell activation and function can, therefore, be evaluated in terms of reorganization and modulation of the cytoskeleton (Hui and Upadhyaya, 2017; Matov, 2024e; Matov, 2024g; Matov, 2024i; Matov and Danuser, 2004; Ponti et al., 2003), and the EB1 comet metrics will be validated by the levels of measured cytokine production. After validation and calibration, the software can be utilized for the discovery of novel drug combinations that allow personalized immunotherapy treatment.

## Discussion

Our analyses underscore the importance of assessing MT dynamics in patient cells by quantitative live-cell assays *ex vivo* in order to anticipate drug resistance in patient tumors. We present new imaging data and measurements of cells expressing SV40, which indicate that its expression in normal epithelium leads to aneuploidy and multiple hallmarks of cancer that are very similar to the aberrations in receptor-negative cancer cells. Our results in the analysis of CTCs and patient-derived organoids before and after drug treatment demonstrate a feasibility for the validation of our computational strategies and translating them to the clinic, which would reduce the time for reviewing samples from hours to a few minutes.

Further, this contribution proposes novel strategies for overcoming drug resistance in cancer by targeting specific MT-regulating genes and we outline examples in immunotherapy. We also present new computational analyses of the effects of docetaxel in cancer cells, which demonstrate that low drug doses are more efficient in affecting the regulation of the MT cytoskeleton and suggest novel treatment approaches that do not rely on killing the cells, but rather on modulating their regulation, as well as a novel computational assay for drug discovery. Doses and schedules of oncology drugs are sometimes inadequately characterized and the “more is better” paradigm is still used for dose selection, despite the recognition of a need for dose optimization (Shah et al., 2021). The changes in MT organization in disease and during treatment, and the changes in MT dynamics are important prognostic and predictive indicators, and we propose to extend our current analysis framework by the utilization of novel artificial intelligence (AI) algorithms, such as foundation models.

The ability of MTs to reorganize and have the dynamic processes at their ends regulated like during normal physiology is essential. When some of the 12 parameters (Matov et al., 2010) of MT dynamic instability are out of their normal range, that is a hallmark of disease. In cancer, there are many examples of faster (Gonçalves et al., 2001; Matov, 2024d) or slower (Galletti et al., 2014b; Matov, 2024f) than normal MT polymerization rates in drug-resistant cells, but there are very little statistics on the remaining 11 parameters (Matov, 2025a). During cell division of cancer cells, a plethora of MT-regulating proteins (Lyle et al., 2009a; Lyle et al., 2009b; Vale, 2003), that do not directly modulate the rates of polymerization, affect changes in the organization of the spindle poles (Matov, 2025b). MTs face the spindle poles with their minus ends and there is little information, in terms of systematic measurements, in regard to parameters like periods of MT pausing and rates of depolymerization (or the frequencies of switching between these states) in patient cells (Matov, 2024g).

### Targeting RAS mutations

*Ras* genes are well established as the most frequently mutated oncogenes in human cancer, but remain difficult to target (Stephen et al., 2014). Almost one in five of all human cancers were shown to harbor a RAS alteration and KRAS represents the majority (more than 80%) of mutated RAS in solid tumors, yet with the approved KRAS^G12C^(OFF)-only inhibitors only 12% of KRAS-mutated tumors can be directly targeted and 36% of patients treated with sotorasib, for instance, experience either primary resistance or early disease progression after less than 3 months (Singhal et al., 2024). *Ras* is activated by GTP loading and deactivated upon GTP hydrolysis to GDP, that is, (ON) and (OFF) states, respectively. In epithelial tumor cells, the concentration of GTP is between 0.1 and 1 mM (Kopra et al., 2023), while the affinity of RAS for GTP is within the pM range (Bos, 2018). Therefore, a strategy of limiting the available pool of GTP molecules is applicable when the GTP concentration is significantly decreased, for example, in quiescent cells that are starved (Matov, 2024d). To this end, drug combinations that maintain MT polymerization (like the taxanes and epothilones) and inhibit switches to depolymerization or pausing could be utilized together with compounds that induce an activity of MT-severing enzymes (the ATPases katanin, spastin, and fidgetin) (Kuo and Howard, 2021). MT severing proteins catalyze tubulin exchange along the MT lattice and, thus, patch up the new-formed GDP-bound MT ends with GTP-bound tubulin (Vemu et al., 2018). Phosphorylation of Op18 (Cassimeris, 2002; Tournebize et al., 1997) and/or the inhibition of KIF2C (Manning et al., 2007) would reduce the frequency of catastrophe as well as increase the sensitivity to taxane treatment (Mistry and Atweh, 2006; Pao et al., 2012). Treatment with the histone deacetylase trichostatin A (TSA) would increase the MT polymerizing plus pausing times (that is, the overall time MTs spend before switching to depolymerization) (Matov, 2025a). This way, by considering the combined effects on MT dynamics induced, we could identify a drug combination that maximizes the availability of polymerizing MT ends that recruit GTP molecules and minimize their availability to the RAS GTPases. The quantitative live-cell imaging approach for the evaluation of MT dynamics we present here can, thus, help refine and personalize the treatment regimen based on the *ex vivo* analysis of patient-derived cells.

In mCRPC, 40% of patients progress by 18 weeks and some patients suffer all the toxicities of docetaxel with limited benefit. P38 MAPK activity is associated with docetaxel resistance and inhibition of p38 MAPK is synergistic with docetaxel *in vitro* and *in vivo*. The mechanism remains unclear, but may involve destabilization of MTs (Li and Febbo, 2013). RASopathies are a collection of conditions caused by hyperactivation of the MAPK pathway (McCormick, 2024). MAPK-catalyzed phosphorylation of MAP2 and MAP4 reduces the rates of MT polymerization (Hoshi et al., 1992). BBO-10203, a first-in-class, selective blocker of the PI3Kα:RAS interaction, inhibits tumor growth without inducing hyperglycemia (Sinkevicius et al., 2025). It specifically binds to the RAS-binding domain of PI3Kα and prevents its activation by HRAS, NRAS, and KRAS (Simanshu et al., 2025). PI3K activation on endosomes is dependent upon the interaction of PI3Kα with MAP4 (Batrouni and Baskin, 2020), which regulates the localization of membrane vesicle-associated PI3Kα to MTs (Thapa et al., 2020). Quantitative live-cell image analysis of MT dynamics will facilitate the measurements of the cellular effects of BBO-10203 and other novel therapies targeting both the active (ON) and inactive (OFF) conformations of mutant KRAS, such as BBO-8520 (monotherapy or in combination with pembrolizumab) (Maciag et al., 2025; Solomon et al., 2025) and BBO-11818 (Espinosa et al., 2025), in the context of describing and anticipating the emerging mechanisms of acquired drug resistance in patient-derived cells.

### Targeting KIF18A

Kinesins conventionally act as molecular motor proteins that translocate along MTs (Vale et al., 1996). However, several kinesins also control MT dynamics. The kinesin-8 motors, KIF18A in human and Kip3p in yeast (Mayr et al., 2007; Varga et al., 2006), have a unique combination of MT plus end-directed motor protein and MT plus end (unlike KIF2C/MCAK, which localizes at both MT ends (Desai et al., 1999; Hunter et al., 2003)) depolymerase activities. These activities facilitate the positioning of the chromosomes at the metaphase plate (Stumpff et al., 2008) and the mitotic spindle at the cell cortex. Receptor-negative breast tumor cells form large rotating spindles with long astral MTs (Matov, 2024f) and the kinesin-8 motors can depolymerize such long MTs faster than short MTs (Mayr et al., 2007; Varga et al., 2006). The depletion of KIF18A induces aberrantly long mitotic spindles, resulting in the activation of the Mad2-dependent SAC as the chromosomes completely fail to align at the spindle equator (Mayr et al., 2007). Targeting KIF18A with VLS-1272 (Phillips et al., 2025), a novel KIF18A inhibitor, can, thus, have a clinical utility in TNBC and the quantitative live-cell imaging approach for the evaluation of MT dynamics we present here can elucidate the mechanism of action.

Cells exhibiting chromosomal instability are vulnerable to KIF18A inhibition (Cohen-Sharir et al., 2021; Marquis et al., 2021; Payton et al., 2024; Quinton et al., 2021; Research Briefing, 2024). VLS-1488, another KIF18A inhibitor, has demonstrated a favorable safety profile at clinically active doses, with tumor shrinkage in patients with advanced cancers (Dumbrava et al., 2025; Gaillard et al., 2024). As KIF18A plays a crucial role in regulating MT dynamics during mitosis, the *ex vivo* MT dynamics analysis of patient cells can facilitate patient stratification and elucidate the emerging mechanisms of drug resistance in clinical trials of KIF18A inhibitors, such as VLS-1488, sovilnesib (Belmontes et al., 2023; Govindan et al., 2021), and ATX-295 (Lynes et al., 2025). Targeting KIF18A has also been shown to enhance the activation of cytotoxic T cells and PD-1 blockade efficiency in tumors with chromosomal instability phenotype (Liu et al., 2025), which indicates modulation of MT dynamics and suggests the utilization of our quantitative live-cell imaging approach to optimize immunotherapy.

### Dose-adjusted immunotherapy

There are therapies that include MT targeting and immunotherapy for which the pharmacodynamic dose-adjustments are particularly important to treat, for instance, highly proliferative lymphomas. The treatment of drug-naïve high-risk Burkitt lymphoma or aggressive diffuse large B-cell lymphoma with *Myc* gene rearrangement (Scott et al., 2018), the dose-adjusted R-EPOCH regimen consists of six drugs – etoposide, prednisone, vincristine, cyclophosphamide, doxorubicin, and rituximab (Chamuleau et al., 2023; Dunleavy et al., 2018). Similarly, the R-CODOX-M regimen consists of rituximab, cyclophosphamide, cyclophosphamide, vincristine, doxorubicin, and methotrexate (Chamuleau et al., 2023). We propose to utilize our quantitative live-cell imaging approach for the evaluation of MT dynamics in patient-derived lymphocytes *ex vivo* and optimization of the dose for each of the drugs.

Some of the dynamic changes in disease at MT plus ends during stages of pausing and depolymerization, or at MT minus ends, can be deduced by genetic sequencing data. However, in many cases these changes involve a variety of mechanisms that cannot be easily deduced and only quantitative live-cell imaging could deliver precise information on the main induced phenotype. In this context, methodology for visualizing MTs while they pause and depolymerize will allow us to perform exact measurements of these stages of MT homeostasis. This approach will improve our understanding not only of the effects of systemic therapy in cancer, but also of immunotherapy, where the MT cytoskeleton is intimately involved during the formation of the immunological synapse and the release of cytotoxic lysosomes (Blas-Rus et al., 2017; Matov, 2024g). Currently, the low rates of success in the treatment of solid tumors prevent immunotherapy from becoming the standard of care for tumors of epithelial origin (Curti, 2025). For these reasons, new clinical research, such as the analysis of live patient cells and their response to tentative regimens *ex vivo*, and the collection of longitudinal datasets (Matov, 2024k) for each patient is of essence.

Treatment with ivosidenib (Merchant et al., 2019) leads to durable remissions in *IDH1*-mutated relapsed or refractory AML (DiNardo et al., 2018). Mutant *IDH1* decreases NAD^+^ levels and a depletion of NAD^+^ is specific for *IDH1* mutant cancers (Tateishi et al., 2015). We have demonstrated that NAD^+^ increases the rates of MT turnover (polymerization and depolymerization) in cancer cells and leads to resistance to MT-destabilizing drugs (Harkcom et al., 2014) and conversely, lowering the levels of NAD^+^ can be linked to attenuation of MT turnover rates and susceptibility to tubulin inhibitors like vinblastine (Matov, 2024f). Novel combination therapies for the treatment of advanced AML that also include immunotherapy drugs can be evaluated in patient-derived cells with the computational assay we present here.

### Toxicokinetics and pharmacogenomics

Novel drugs can be evaluated with our computational approach during drug development in combination with a benchmark dose gene expression analysis (EFSA Scientific Committee et al., 2022). The use of gene expression signatures to classify compounds, identify efficacy or toxicity, and differentiate close analogs depends on the sensitivity of the method to identify modulated genes. Ligation-based targeted whole transcriptome expression profiling assays are able to determine whether previously unreported compound-responsive genes could be identified and incorporated into a compound-specific signature – for example, related to the activity of the histone deacetylase inhibitor TSA (Yeakley et al., 2017), which affects multiple parameters of MT dynamics (Matov, 2025a). Our quantitative computer vision measurements in living cells can be considered together with transcriptomics datasets and concentration-response modeling (Harrill et al., 2021). This approach can evaluate the transcriptional biological pathway altering concentrations (Judson et al., 2011) and the toxicokinetics of novel compounds. Dose-response curves indicate when a drug concentration induces drug efficacy or triggers toxicity (Ramaiahgari et al., 2019) based on gene expression and the metabolism induced. The analysis will elucidate the specific changes induced by the drug doses applied to patient cells *ex vivo* and relate the cytoskeletal dynamics metrics we compute to pharmacogenomics during drug discovery. This way, we can identify dependencies between each parameter of MT and actin dynamics, and the metabolic changes in patient cells. This approach will allow us to dissect the differences in drug action between drugs that bind to the same domain, for example, as taxanes or vinca alkaloids.

### Foundation models for drug discovery

We can characterize the progression of cancer as a heterogeneous directed Markov chain with absorbing states (Bremaud, 1999; Matov, 2024i) and modeling of sequential input data as a Markov process is an intuitive way to utilize large language models (LLMs) (Ren et al., 2024) because of the Markovianity of natural languages (Shannon, 1951). To this end, we will retrain a transformer network (Vaswani et al., 2017) with a new set of tokens – with genetic mutations rather than words and with tumor burden values rather than sentences of human speech, that is, we will retrain a foundation model with disease progression datasets. Additional tokens will be the texture metrics of the organoids such as shape descriptors and other morphology data (Boland and Murphy, 2001; Matov, 2024c; Matov, 2024i; Murphy et al., 2003) as well as metrics regarding DNA fragment length patterns before and after disease diagnosis (Cristiano et al., 2019; Matov, 2024a), and changes in the regulation of small RNAs (Matov, 2024b; Matov, 2024k; Zomer et al., 2010). Our analysis will be sensitive to detecting metabolic changes, such as ferroptosis in immune cells (Chen et al., 2021), characteristic for pre-clinical and early-stage disease (Matov, 2024a; Matov, 2024c).

The embeddings will include datasets of cytoskeletal dynamics before and after treatment with different drugs. We have generated several thousand time-lapse microscopy movies with about 2,000-10,000 trajectories consisting of about 3-20 fluorescent features (either MT comets or tubulin and actin speckles) linked in each trajectory (Brennan et al., 2008; Galletti et al., 2014b; Gatlin et al., 2010; Gatlin et al., 2009; Harkcom et al., 2014; Matov, 2024d; Matov, 2024e; Matov, 2024f; Matov et al., 2010; Matov et al., 2016; Matov et al., 2015; Thoma et al., 2010). These are about 60 million data points, but many more existing movies/data can be obtained from our academic collaborators. As we have analyzed the live-cell datasets, the preliminary computer vision analysis has already been done and these results have been validated and published, which will facilitate the training and benchmarking.

Such AI analysis will allow retraining of a generative transformer network to classify each new patient sample in the context of clinical diagnosis and further make predictions regarding disease prognosis. The predictive analysis will allow training the network to classify as sensitive or resistant each new tumor and further identify subclasses with different mechanisms of resistance. Our hypothesis is that a dependency exists between the effects of (and susceptibility to) tubulin inhibitors and those of immune cells, and when it is exploited, it might cause the death of patient tumor cells.

## Materials and methods

### Coverslip processing

Peripheral blood previously drawn from mCRPC patients (the samples were de-identified) receiving docetaxel-based first line chemotherapy was thawed and processed using ficolling (Ficoll-Paque®) to separate cells in the buffy coat from the blood. The cells were isolated by centrifugation, fixed in phenol, and blocked on 8 mm coverslips. In brief, the ficolling method, through centrifugation, isolates peripheral blood mononuclear cells and plasma from granulocytes and erythrocytes. The mononuclear layer contains lymphocytes, monocytes, and CTCs. These cells are plated onto an 8 mm coverslip and subsequently stained for biomarkers (PSMA, Tyr-tubulin, AR, DAPI, and CD45). PSMA labeling was done using ^177^Lu-J591, anti-PSMA monoclonal antibody J591 (Matov, 2024h).

### Sample processing

Retroperitoneal lymph node organoid whole genome sequencing (736 ng DNA) was done at the UCSF Core Facilities.

### Bioinformatics analysis

Sequence reads were trimmed using Trimmomatic 0.36 (Bolger et al., 2014). We used hg38 genome as reference and aligned the reads with BWA-MEM version 0.7.15 (Li and Durbin, 2010). For variant calling, we utilized SAMtool (Li et al., 2009), BCFtools (Li, 2011), Ensembl Variant Effect Predictor v86 (McLaren et al., 2016), VCFtools (Danecek et al., 2011), and ANNOVAR (Wang et al., 2010). To run the analysis, we installed a virtual machine (Oracle VM Virtual Box 5.0.6) and Ubuntu 12.04.5 on a Windows server with Intel Xeon E5-1620 processor with four dual cores and a solid-state drive. For the alignment step, we connected to a high performance computing cluster (Mount Moran, 2016).

### Image analysis

Computational fitting of Gaussian kernels to MT polymerization speed datasets was performed using the *lsqnonlin* function in Matlab, which allowed to determine the mean value and standard deviation for each condition (Matov et al., 2006). All image analysis programs for EB1 (or EB3) comet detection, MT dynamics, and graphical representation of the results were developed in Matlab and C/C++. The computer code is available for download at: https://github.com/amatov/ClusterTrackTubuline. All image analysis programs for PSMA, α-tubulin, DAPI, and CD45 segmentation as well as 3D AR and DAPI segmentation, and graphical representation of the results were developed in Matlab and C/C++. The wavelet transform method used, spotDetector, was described and validated in (Olivo-Marin, 2002) and the unimodal pixel intensity thresholding in (Rosin, 2001). We identified the CTC areas as connected-component labeling pixel lists in the epithelial tumor imaging channel (such as PSMA or occasionally CK) for which in the nuclear imaging channel there is a DAPI stain with a statistically representative size and a circular shape. At the same time, we required that these cells are negative in the CD45, that is, an absence of a leukocyte marker. Similarly, to detect neutrophils and lymphocytes, we identified clusters of bright pixels in the CD45 channel for which a nuclear area is detected in the DAPI channel and the epithelial stain is not present. On multiple occasions, we detected double-positive (PSMA+/DAPI+/CD45+ or CK+/DAPI+/CD45+) and double-negative (PSMA-/DAPI+/CD45- or CK-/DAPI+/CD45-) cells, which we classified in separate bins. Drug-induced MT bundles were evaluated in terms of thickness and texture parameters in 63× magnification images. The detection of tyrosinated tubulin in 4× magnification images was considered to be a marker of drug sensitivity. We analyzed the cellular localization of AR in 3D image stacks (Matov, 2024b), in which we performed segmentation in individual 2D images and reconstructed the volume, and classified the CTCs as sensitive to drug treatment if the AR is within the volume of the nucleus and resistant if the AR is in the cytoplasm. The computer code is available for download at: https://github.com/amatov/SegmentationBiomarkerCTC, https://github.com/amatov/ResistanceBiomarkerAnalysis, and https://github.com/amatov/AntibodyTextureMorphology.

### Statistical analysis

Statistical tests were performed in Matlab. We performed Kolmogorov-Smirnoff statistics (Kolmogorov, 1933) to compare higher-order moments of the underlying MT parameter per-track distributions and permutation *t*-test to extract information on shifts in median values of polymerization parameters over a population of cells. In brief, for each condition, multiple videos containing one or two cells were compared. Before merging the data for further statistical analysis, we determined the video-to-video variation in the distributions of MT parameters using the Kolmogorov-Smirnov test (Kolmogorov, 1933). Distributions were subsampled to 400 values to avoid hypersensitivity of the Kolmogorov-Smirnov statistics. Videos passing the consistency test were included in the final datasets. Statistical comparison of MT dynamics between different conditions was accomplished in two steps. First, we examined whether the different conditions would profoundly change the distribution of each parameter, which would indicate an entirely different state of MT regulation between conditions. To test this possibility, we aligned the distributions of compared conditions at their respective per-track mean values and then applied the nonparametric Kolmogorov-Smirnov test to determine whether the two distributions are identical. The distributions were randomly subsampled to 400 values. The subsampling was repeated 200 times, and the final p-values were derived by averaging. In a second step, we compared the shifts in parameter mean values between different conditions. To achieve this, we calculated the per-cell means for each parameter. Then we tested normality of the distribution of the means and applied a test to define the significance of parameter changes between conditions (mean ± SEM of cell-to-cell variation). This test assumes that the cell-to-cell variation is the single biggest source of variation in the mean parameter value of a condition. None of the parameters of MT dynamics followed a Gaussian distribution. To determine the significance of differences in the mean values between parameters under different experimental conditions, we applied a non-parametric permutation *t*-test with 400 repetitions (Hesterberg et al., 2005), which compares the bootstrapped distributions of a condition 1 versus condition 2. This test does not make any assumption about the characteristic of the tested distribution and, thus, is appropriate for application to distributions that are far from being normally distributed. In brief, for each condition 400 values are bootstrap-sampled from the data. In agreement with the central limit theorem (Laplace, 1810), the two bootstrapped distributions always follow a Gaussian distribution and, thus, could be analyzed for differences using a regular Student’s *t*-test.

### Live-cell microtubule imaging

DU145 cells were transduced with plasmid encoding EB1ΔC-2xEGFP and EB1 comets in live cells were imaged by spinning-disc confocal microscopy using a 100× magnification oil immersion 1.49 NA objective as previously described (Gierke and Wittmann, 2012) and tracked using our image analysis algorithm (Matov et al., 2010). EB1 transiently binds to growing MT plus ends (Akhmanova and Steinmetz, 2008), generating a punctate pattern of EB1ΔC-2xEGFP comets throughout the cell. The exponential decay of available binding sites results in the characteristic comet-like fluorescence profiles of EGFP-tagged end binding proteins (“comets”). DU145-GFP and DU145-ERG cells were treated with 5 nM and 10 nM docetaxel for 1 h. To test for statistically significant shifts in the distributions of MT growth rates obtained following the tracking of 200,000 EB1ΔC-2xEGFP comets, we performed per-MT track and per-cell analysis, and compared the average EB1 speeds for 87 docetaxel-treated cells (see Statistical Analysis). hTERT-immortalized retinal pigment epithelial (RPE-NEO) cells expressing very low levels of MYC and RPE-MYC cells engineered to constitutively overexpress MYC were cultured and imaged as previously described (Rohrberg et al., 2020). Receptor TNBC MDA-MB-231 cells were cultured and imaged as previously described (Matov, 2024f). We imaged EB1ΔC-2xEGFP-expressing organoids (Koo et al., 2013) by time-lapse spinning disk confocal microscopy using a long working distance 60× magnification water immersion 1.45 NA objective. We acquired images every half a second for a minute (that is, 120 images per dataset) to collect datasets without photobleaching (Gierke and Wittmann, 2012). 

### Microscopy imaging

The type of microscopy used to image fixed cells was spinning-disc confocal microscopy with a 10× magnification 0.3 NA objective. The acquisition was accomplished using an ORCA-Flash 4.0 (6.5 µm/pixel) camera.

Organoids were imaged using transmitted light microscopy at 4× magnification and phase contrast microscopy at 20× magnification on a Nikon Eclipse Ti system with camera Photometrics CoolSnap HQ2.

Organoid long-term imaging was done over 72 h by taking an image every 10 min at 20× magnification.

For imaging of patient CTCs, the type of microscopy used was widefield epi-fluorescence with 10× and 63× magnification objectives. The acquisition was accomplished using an ORCA-Flash 4.0 (6.5 µm/pixel) camera.

### Cell culture

Primary and retroperitoneal lymph node metastatic prostate tumor and sternum metastatic rectal tumor tissues were dissociated to single cells using modified protocols from the Witte lab (Goldstein et al., 2011). Organoids were seeded as single cells in three 30 µL Matrigel drops in 6-well plates. Organoid medium was prepared according to modified protocols from the Clevers lab (van de Wetering et al., 2015) and the Chen lab (Gao et al., 2014).

To obtain a single cell suspension, tissues were mechanically disrupted and digested with 5 mg/mL collagenase in advanced DMEM/F12 tissue culture medium for several hours (between 2 and 12 h, depending on the biopsy or resection performed). If this step yielded too much contamination with non-epithelial cells, for instance during processing of primary prostate tumors, the protocol incorporated additional washes and red blood cell lysis (Goldstein et al., 2011). Single cells were then counted using a hemocytometer to estimate the number of tumor cells in the sample, and seeded in growth factor-reduced Matrigel drops overlaid with PC medium (Gao et al., 2014). With radical prostatectomy specimens, we had good success with seeding 3,000 cells per 30 μL Matrigel drop, but for metastatic samples organoid seeding could reliably be accomplished with significantly less cells, in the hundreds. To derive organoids from patient CTCs, liquid biopsy samples of 40 mL peripheral blood would be collected, processed, and plated in a Matrigel-collagen-fibronectin matrix to form organoids similarly to the metastatic BC organoids we cultured from mouse CTCs (Matov, 2024d).

## Data Availability

The datasets presented in this study can be found in online repositories. The names of the repository/repositories and accession number(s) can be found below: https://www.ncbi.nlm.nih.gov/sra/SRX28551163.

## Glossary

ADP: adenosine diphosphate
AI: artificial intelligence
AML: acute myeloid leukemia
AR: androgen receptor
ARF: ADP-ribosylation factor
ATPase: adenosine triphosphatase
BC: breast cancer
BRCA2: breast cancer type 2 susceptibility protein
CD45: protein tyrosine phosphatase, receptor type, C
CIN: chromosomal instability
CK: cytokeratin
CLASP2: cytoplasmic linker-associated protein, 2
CLIP170: cytoplasmic linker protein, 170
CTC: circulating tumor cell
CYPA: cyclophilin A
DAPI: 4′,6-diamidino-2-phenylindole
EB1/3: microtubule end binding protein, 1/3
EB1ΔC: EB1 construct truncated at amino acid 248 that does not interact with other proteins at the microtubule end
EGFP: enhanced green fluorescent protein (2xEGFP indicates two molecules)
EpCAM: epithelial cell adhesion molecule
ERG: erythroblast transformation-specific family-related gene
FA: focal adhesion
FGF: fibroblast growth factor
FN: false negative
FP: false positive
F-actin: filamentous actin
GAP: GTPase-activating protein
GDP: guanosine diphosphate
GEDI: geometrically enhanced differential immunocapture
GTPase: guanosine triphosphatase
GSK3β: glycogen synthase kinase 3, beta isoform
GTP: guanosine triphosphate
HIFα: hypoxia-inducible factor, alpha subunit
LLM: large language model
Mad2: mitotic arrest deficient protein, 2
MAP: microtubule-associated protein
MAPK: mitogen-activated protein kinase
MAP1B: microtubule-associated protein, 1B
MAP2: microtubule-associated protein, 2
MAP4: microtubule-associated protein, 4
MCAK/KIF2C: mitotic centromere-associated kinesin
mCRPC: metastatic castrate-resistant prostate cancer
MT: microtubule
NAD^+^: nicotinamide adenine dinucleotide, oxidized
NK: natural killer cell
Op18: oncoprotein, 18 (stathmin)
PC: prostate cancer
PI3Kα: phosphoinositide 3-kinase, alpha subunit
PLK1: polo-like kinase, 1
PSMA: prostate-specific membrane antigen
RIT1: RAS-like without CAAX 1
ROCK: Rho-associated protein kinase
RT-PCR: reverse transcriptase polymerase chain reactions
SAC: spindle assembly checkpoint
Skp2: S-phase kinase associated protein, 2
SV40: Simian virus, 40
TCI: tri-complex inhibitor
TIL: tumor-infiltrating lymphocyte
TMPRSS2: transmembrane protease, serine 2
TN: true negative
TNBC: triple-negative breast cancer
TP: true positive
TSA: trichostatin A
pVHL: von Hippel-Lindau protein
