# Supplementary material for: Microtubule regulation in cancer cells
Source: Front Cell Dev Biol. 2026 Aug 21;13:1677302. doi: 10.3389/fcell.2025.1677302 (PMC13543818; doi:10.3389/fcell.2025.1677302)
Supplement: Supplementary file 1 [file Supplementaryfile1.doc]

**SUPPLEMENTARY MATERIALS**

Table S1 – EB1 comet speeds in metaphase and interphase breast cancer cells.

Table S2 – MYC overexpression reduces MT turnover rates in interphase RPE cells.

Table S3 – 10 nM docetaxel does not reduce MT turnover rates in DU145 cells.

Video 1 – Rotating spindle in TNBC after MYC knockdown.

Video 2 – Rotating and misplaced spindle in TNBC after MYC knockdown.

Video 3 – Rotating spindle in an HBL-100 breast epithelial cell expressing SV40 large T antigen.

Video 4 – Misplaced spindle in an HBL-100 breast epithelial cell expressing SV40 large T antigen.

Video 5 – Unfocused spindle poles in an HBL-100 breast epithelial cell expressing SV40 large T.

Video 6 – Metaphase RPE-MYC spindle with detected EB1 comets.

Video 7 – Interphase RPE-MYC cells with detected EB1 comets.

Video 8 – Rotating spindle in an RPE-MYC cell.

Video 9 – Unfocused spindle pole in an RPE-MYC cell.

Video 10 – Tri-polar spindle in an RPE-MYC cell.

Video 11 – Primary colon cancer organoid treated with 2 nM vinorelbine.

| Cells | Cell cycle | MT tip speed | Videos | MT tracks |
| --- | --- | --- | --- | --- |
| MDA-MB-231 | Interphase | 18.5 µm/min | 3 | 12,962 |
| LY2 | Interphase | 16.2 µm/min | 3 | 4,213 |
| ZR-75-B | Interphase | 9.4 µm/min | 3 | 2,536 |
| HCC-1143 | Interphase | 13.5 µm/min | 3 | 4,526 |
| HCC-1428 | Interphase | 11.5 µm/min | 3 | 7,886 |
| HCC-3153 | Interphase | 13.1 µm/min | 3 | 8,649 |
| MDA-MB-231  Rotating | Metaphase | 18.8 µm/min | 2 | 2,955 |
| MDA-MB-231 | Metaphase | 13.5 µm/min | 2 | 2,652 |
| HBL-100 | Metaphase | 17.8 µm/min | 2 | 2,019 |
| LY2 | Metaphase | 13.5 µm/min | 1 | 871 |
| ZR-75-B | Metaphase | 14.2 µm/min | 1 | 1,109 |
| HCC-1143 | Metaphase | 12.3 µm/min | 1 | 542 |
| HCC-1428 | Metaphase | 16.5 µm/min | 1 | 1,187 |

**Table S1.** **EB1 comet speeds in breast cancer cell lines.** MDA-MB-231 (receptor negative), HCC-1143 (receptor negative), HCC-3153 (receptor negative), ZR-75-B (estrogen receptor-positive), LY2 (progesterone receptor-positive), HCC-1428 (estrogen receptor-positive, progesterone receptor-positive), HBL-100 (breast epithelium with expressed Simian virus 40 large T antigen). Computational analysis of MT dynamics was performed using ClusterTrack (Matov et al., 2010). Each movie consists of 125 image frames taken 0.5 seconds apart. Conditions for which the metaphase MT polymerization rates are different with statistical significance (p < 0.05) have the EB1 comet speed numbers shown in red (higher rates).

| Cells | Cell cycle | MT tip speed | Videos | MT tracks |
| --- | --- | --- | --- | --- |
| RPE-NEO | Metaphase | 16.1 µm/min | 8 | 14,241 |
| RPE-MYC | Metaphase | 16.0 µm/min | 12 | 15,088 |
| RPE-NEO | Anaphase | 13.2 µm/min | 4 | 7,559 |
| RPE-NEO | Interphase | 13.1 µm/min | 3 | 3,929 |
| RPE-MYC | Interphase | 10.9 µm/min | 3 | 3,011 |

**Table S2. MYC overexpression reduces MT turnover rates in interphase RPE cells.** The hTERT-immortalized retinal pigment epithelial (RPE-NEO) cell line has very low levels of MYC. RPE cells were engineered to constitutively overexpress MYC (RPE-MYC). Computational analysis of MT dynamics was performed using ClusterTrack (Matov et al., 2010). The RPE-MYC cells had reduced numbers of growing MTs both during mitosis and interphase, and also decreased MT polymerization rates during interphase. Each movie consists of 125 image frames taken 0.5 seconds apart (for EB1 comet detection in a metaphase and an interphase cell, see Vid. 6 and Vid. 7, respectively). The condition for which the interphase MT polymerization rates are different with statistical significance (p < 0.05) has the EB1 comet speed numbers shown in blue (lower rates).

| Cells | Docetaxel | MT tip speed | Videos |
| --- | --- | --- | --- |
| DU145-GFP | - | 11.4 µm/min | 12 |
| DU145-ERG | - | 9.6 µm/min | 15 |
| DU145-GFP | 1 nM | 8.1 µm/min | 10 |
| DU145-ERG | 1 nM | 10.3 µm/min | 14 |
| DU145-GFP | 5 nM | 9.5 µm/min | 13 |
| DU145-ERG | 5 nM | 10.7 µm/min | 17 |
| DU145-GFP | 10 nM | 11.0 µm/min | 12 |
| DU145-ERG | 10 nM | 11.2 µm/min | 14 |
| DU145-GFP | 10 nM | 10.9 µm/min | 16 |
| DU145-ERG | 10 nM | 10.3 µm/min | 15 |

**Table S3.** **10 nM docetaxel does not reduce MT turnover rates in DU145 cells.** DU145 prostate cancer parental cell line and an ERG-overexpressing derivative cell line that shows a 20-fold increased docetaxel resistance were treated with 5 nM and 10 nM docetaxel (the data for 1 nM docetaxel was taken from (Galletti et al., 2014)). Computational analysis of MT dynamics was performed using ClusterTrack (Matov et al., 2010). Each movie consists of 125 image frames taken 0.5 seconds apart. Conditions for which the MT polymerization rates are different with statistical significance (p < 0.05) have the EB1 comet speed numbers shown in blue (lower rates).

Galletti, G., A. Matov, H. Beltran, J. Fontugne, J. Miguel Mosquera, C. Cheung, T.Y. MacDonald, M. Sung, S. O'Toole, J.G. Kench, S. Suk Chae, D. Kimovski, S.T. Tagawa, D.M. Nanus, M.A. Rubin, L.G. Horvath, P. Giannakakou, and D.S. Rickman. 2014. ERG induces taxane resistance in castration-resistant prostate cancer. *Nature communications*. 5:5548.

Matov, A., K. Applegate, P. Kumar, C. Thoma, W. Krek, G. Danuser, and T. Wittmann. 2010. Analysis of microtubule dynamic instability using a plus-end growth marker. *Nature Methods*. 7:761- 768.
